# The role of individual variability on the predictive performance of machine learning applied to large bio-logging datasets

**DOI:** 10.1038/s41598-022-22258-1

**Published:** 2022-11-17

**Authors:** Marianna Chimienti, Akiko Kato, Olivia Hicks, Frédéric Angelier, Michaël Beaulieu, Jazel Ouled-Cheikh, Coline Marciau, Thierry Raclot, Meagan Tucker, Danuta Maria Wisniewska, André Chiaradia, Yan Ropert-Coudert

**Affiliations:** 1 Centre d’Etudes Biologiques de Chizé, UMR7372 CNRS - La Rochelle Université, 405 Route de Prissé La Charrière, 79360 Villiers-en-Bois, France; 2 British Antarctic Survey, High Cross, Madingley Road, Cambridge, CB3 0ET UK; 3 German Oceanographic Museum, Stralsund, Germany; 4 Institut de Recerca de la Biodiversitat (IRBio) and Departament de Biologia Evolutiva, Ecologia i Ciències Ambientals (BEECA), Facultat de Biologia, Universitat de Barcelona., Av. Diagonal 643, 08028 Barcelona, Spain; 5 Institut de Ciències del Mar (ICM-CSIC), Departament de Recursos Marins Renovables, Passeig Marítim de la Barceloneta, 37-49, 08003 Barcelona, Spain; 6 Institute for Marine and Antarctic Studies, University of Tasmania, Hobart, TAS 7001 Australia; 7 Institut Pluridisciplinaire Hubert Curien, UMR7178, CNRS-Universite de Strasbourg, Strasbourg, France; 8 Conservation Department, Phillip Island Nature Parks, Cowes, VIC Australia; 9 Sound Communication and Behaviour Group, Department of Biology, University of Southern Denmark, Campusvej 55, DK-5230 Odense M, Denmark

**Keywords:** Behavioural ecology, Ecological modelling, Ecophysiology, Machine learning

## Abstract

Animal-borne tagging (bio-logging) generates large and complex datasets. In particular, accelerometer tags, which provide information on behaviour and energy expenditure of wild animals, produce high-resolution multi-dimensional data, and can be challenging to analyse. We tested the performance of commonly used artificial intelligence tools on datasets of increasing volume and dimensionality. By collecting bio-logging data across several sampling seasons, datasets are inherently characterized by inter-individual variability. Such information should be considered when predicting behaviour. We integrated both unsupervised and supervised machine learning approaches to predict behaviours in two penguin species. The classified behaviours obtained from the unsupervised approach *Expectation Maximisation* were used to train the supervised approach *Random Forest.* We assessed agreement between the approaches, the performance of *Random Forest* on unknown data and the implications for the calculation of energy expenditure. Consideration of behavioural variability resulted in high agreement (> 80%) in behavioural classifications and minimal differences in energy expenditure estimates. However, some outliers with < 70% of agreement, highlighted how behaviours characterized by signal similarity are confused. We advise the broad bio-logging community, approaching these large datasets, to be cautious when upscaling predictions, as this might lead to less accurate estimates of behaviour and energy expenditure.

## Introduction

Extremely large, complex and multidimensional data sets, difficult to handle and analyze with common methodological approaches (so-called big data), are collected and produced by several scientific and industrial fields and require tools to facilitate efficient management, visualization, analysis, validation and interpretation^1^. In biology, big data have emerged with studies on gene and protein sequences, gene expression, evolutionary trees, microscopy images, 3D structures and interaction networks^2–5^. Within global change biology, ecology and remote sensing, the recent use of big data is improving our understanding of interactions between environmental changes and biological systems^6^. In animal movement ecology, using animal-borne tags (bio-logging) can quickly accumulate large amount of data. The quantity of collected data is large due to high sampling resolution, the number of sensors (e.g., a bio-logging tag can include location and pressure sensors, magnetometer, accelerometer and environmental sensors), length of deployment and number of species considered (e.g., more than one year of accelerometer data recordings, decades of data tracking)^7–11^. Tri-axial accelerometers, for example, provide 3-dimensions (called surge, sway and heave) of high-resolution acceleration data to obtain measurements of effort and relative energy expenditure related to different behaviours and activities performed by individual animals^12^. Several variables are calculated from raw acceleration data indicating body orientation and acceleration, such as body pitch, roll, dynamic acceleration for each axis, Vectorial Dynamic Body Acceleration (VeDBA), (see^12,13^ and our Method section for overview). A common proxy of energy expenditure is activity-specific Dynamic Body Acceleration (DBA)^12^. When validated with both direct and indirect measurements of energetic expenditure (heart rates, isotope elimination—Doubly Labelled Water (DLW),—respiratory chambers), DBA can provide estimates of energy consumption (e.g., daily energy expenditure, DEE hereafter) across habitats, generating “energy landscapes”^14–16^. Energetic expenditure attributed to landscape features is currently used to quantify behavioural and fitness responses to habitat availability and human-derived risks at both individual and population level^17–19^.

To extract relevant information from large and complex datasets, the use of Artificial Intelligence tools (e.g. machine learning and deep learning approaches) has steadily grown in importance over the last decade to the point that they are applied in nearly every research field. In the bio-logging field, machine learning has been applied across marine and terrestrial systems to classify types of behaviour performed by animals equipped with fast-sampling accelerometers. Both unsupervised and supervised machine learning approaches, have been used on both data collected in the wild as well as in captivity^20–25^. Usually, unsupervised approaches are used when validation data are not available, in contrast to a supervised approach making use of pre-labelled known behavioural activities recorded. While unsupervised approaches (e.g., Expectation Maximisation, k-means) independently detect behaviours and allow for the detection of unknown behaviours^20,26^, supervised approaches (e.g., Random Forest, Support Vector Machine) are fast and reliable on known behaviours^27,28^. Whilst both approaches have their strengths, they also have their individual and shared weaknesses and can be complementary. Unsupervised machine learning approaches require manual post-labelling, which does not scale well with the exponential increase of data volume. By using pre-labelled training datasets to predict on unknown data, supervised approaches can automatically predict on large volume of data, but are, instead, limited by the information acquired via the training. Integrating both unsupervised and supervised machine learning approaches can make predictions of behaviours across large datasets robust and feasible^29^.

Behavioural validation on elusive species, challenging to monitor in the wild, might be a lacking, or possible only on some behaviours and for short time periods, producing datasets which can be too small to enable robust learning. Moreover, behaviours recorded/characterized in captivity may not be suitable to validate data collected in the wild that are likely to be noisier and/or contain unexpected signals due to the combination of individual variability in movement mechanics, environmental variability, and properties of the medium in which individuals are moving. To address some of these issues, researchers have provided more detailed validation data, recording variation in accelerometer signals of animals walking, for example, on different types of terrain^30^. Depending on the research question tackled, the resolution and type of data collected, number and types of behaviours needed to estimate (few coarse vs many high-resolution behaviours), machine learning approaches might estimate time-activity budgets differently, and hence, lead to differences in DEE values, if DBA values are grouped by activity type (as done in^31,32^, for example). Therefore, in the past few years, this field has taken a more critical look at the available approaches and begun to assess which methods are most appropriate in different scenarios^13,28,33^.

**Figure 1:**
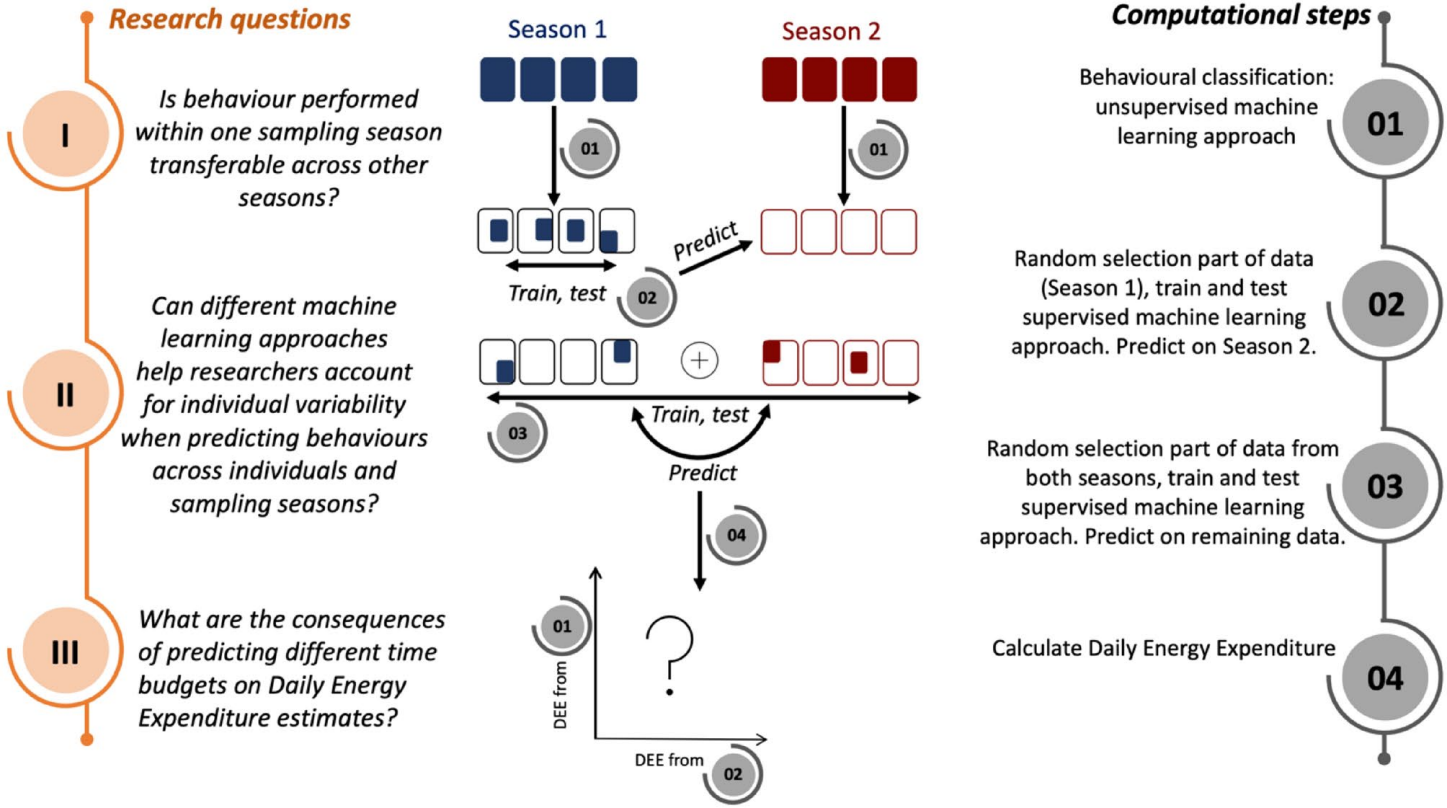
Conceptual overview of research questions and approach followed in this study. Both unsupervised and supervised machine learning approaches are applied on large accelerometer datasets to detect and predict behavioural activities. We quantify the behavioural variation included in the training datasets used for the supervised approach and test their predictive performance. We finally validate DEE estimates resulted from the behavioural activity budgets from the two approaches based on energetic validation obtained on Adélie penguin (*Pygoscelis adeliae)* from Hicks et al.^32^.

As researchers continue to collect data, accelerometer datasets, obtained from large numbers of individuals across years, are inherently characterised by individual and environmental variability^34,35^. The ideal setting for obtaining behavioural validation and data acquisition over a large sample size and sampling years to train machine learning models might not always be feasible in the wild, making inference of behavioural activities and their changes even more challenging. Both unsupervised and supervised machine learning approaches need to deal with individual and environmental variabilities, and the effect of individual variability on the performance of machine learning algorithms is largely unknown^24^. The large volume of complex bio-logging data calls for continuous refinements in analytical methods to make upscaling processes (such as inferring behaviours over larger proportions of novel data compared with training and testing data) reliable, robust and as efficient as possible. Here we explore the feasibility of combining different unsupervised and supervised machine learning methods to address the following issues (Fig. 1): (I) Assuming acceleration data are sampled from individuals belonging to the same population, can behaviours detected across individuals be transferred from one sampling period to another sampling period? (II) is the combination of unsupervised and supervised machine learning a viable approach to include individual variability when predicting behaviours across individuals and sampling seasons? As these approaches might result in differing activity budgets, we also address a third question: (III) what are the consequences of varying budgets for the calculation of DEE?

To explore these broad research questions, we use accelerometer data collected over two breeding seasons from two species of penguins foraging in different environments as a case study: Adélie penguins (*Pygoscelis adeliae*) and little penguins (*Eudyptula minor*). The Adélie penguin is the most abundant seabird species in Antarctica, with around 4 million pairs around the continent^36^. Adélie penguins have high underwater flexibility, frequently diving to shallow depths of 5–10 m, likely to feed just under the ice, but also reaching maximum depths of 115–120 m^37^. This species mainly feed on Antarctic and ice krill (*Euphausia superba* and *E. crystallorophias*) and Antarctic silverfish, *Pleuragramma antarctica*^38^. The Little penguin is distributed in southern Australia and New Zealand and its overall population is estimated to be under 500,000 breeding pairs^39^. Little penguins are high trophic level generalist predators feeding in dynamic coastal environments^40,41^ mainly on sardine (*Sardinops sagax*) and anchovy (*Engraulis australis*), but also red cod (*Pseudophycis bachus)*, barracouta (*Thyrsites atun)* and jack mackerel (*Trachurus declivis)*^42^. Individuals forage in the shallower part of the water column, diving on average within 20 m but also reaching 57 m^43,44^. Both species’ are sensitive to human disturbance and climatic variability^45,46^. The feeding ecology and energetic requirements of these species at fine scales can help us to predict their vulnerability to environmental change. To answer the above-mentioned research questions applied to these two penguin species, we follow four main computational steps (Fig. 1). (1) We first use an unsupervised machine learning approach to quantify the individual variability in behaviour across the datasets within seasons. (2 and 3) By randomly selecting parts of the datasets, we then include such variability in training datasets fed to a supervised approach and test its predictive performance and agreement with the unsupervised approach. For Adélie penguins, for which energetic validation is available^32^, we compare DEE estimates (4), resulting from the behavioural activity budgets estimated by both unsupervised and supervised machine learning approaches.

## Results

The two studied species feed within the water column, hence temperature-depth data were also recorded along with acceleration data and processed (see Methods: *Data Preparation*). The same set of variables were used for both species: VeDBA, pitch, the standard deviation of raw heave at 2 s and change in depth for detecting diving behaviours; VeDBA, pitch, the standard deviation of raw heave at 10 s and the standard deviation of roll at 30 s for detecting subsurface and land/ice behaviours (SI Appendix Table S1). The unsupervised machine learning algorithm, *Expectation Maximisation* (EM), was run first (Fig. 1), followed by the supervised machine learning algorithm *Random Forest*.

### Unsupervised machine learning approach

**Figure 2:**
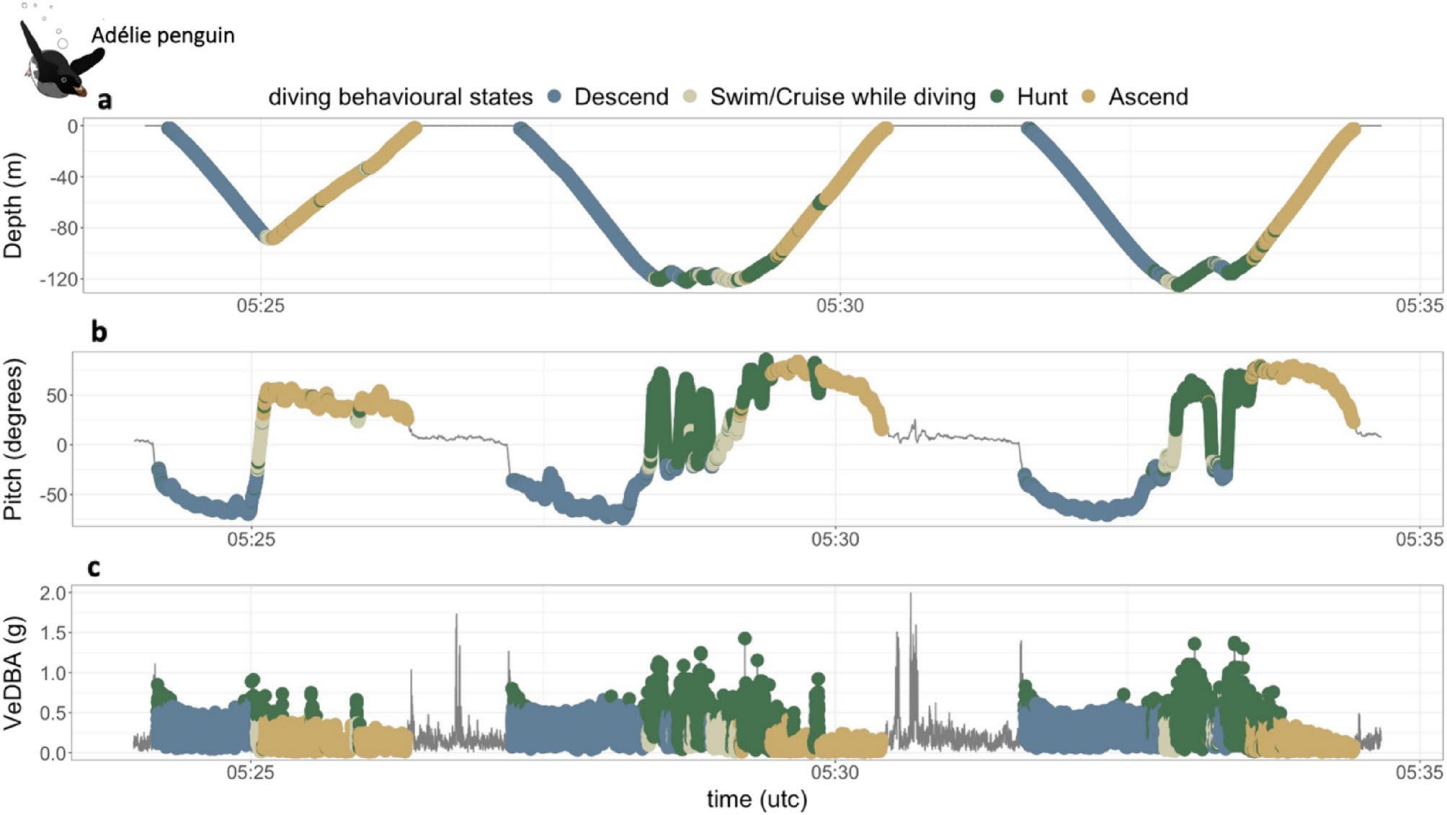
Example of behavioural classification in Adélie penguins (*Pygoscelis adeliae)* while diving. (**a**) diving profiles, (**b**) Pitch (degrees) indicating body posture while diving, (**c**) Vectorial Dynamic Body Acceleration (VeDBA, g) indicating overall acceleration while moving underwater. Colours indicate the behavioural states identified while diving.

Twelve behavioural classes were identified for Adélie penguins. Behaviours such as “descend”, “ascend”, “hunt” and “swim/cruise” were identified from the diving subsets, describing activities performed while diving (Fig. 2). For *Season 1*, we identified an additional swimming behaviour, similar to “swim/cruise”, named “swim/cruise type 2” in 5 foraging trips (out of 107) (SI Appendix Fig. S1). The same additional swimming behaviour was also found in 3 foraging trips (out of 53) in *Season 2*. On land/ice, we identified behaviours such as “walking”, “standing”, “lying down/toboggan” and “preen/high flap on land/ice” (SI Appendix Fig. S2). Finally at the sub-surface/air interface, we identified “slow swim”, “swim/porpoise”, while moving horizontally at the sub-surface, and “preen/high flap on water” behaviours (SI Appendix Figs. S3 and S4).

**Figure 3:**
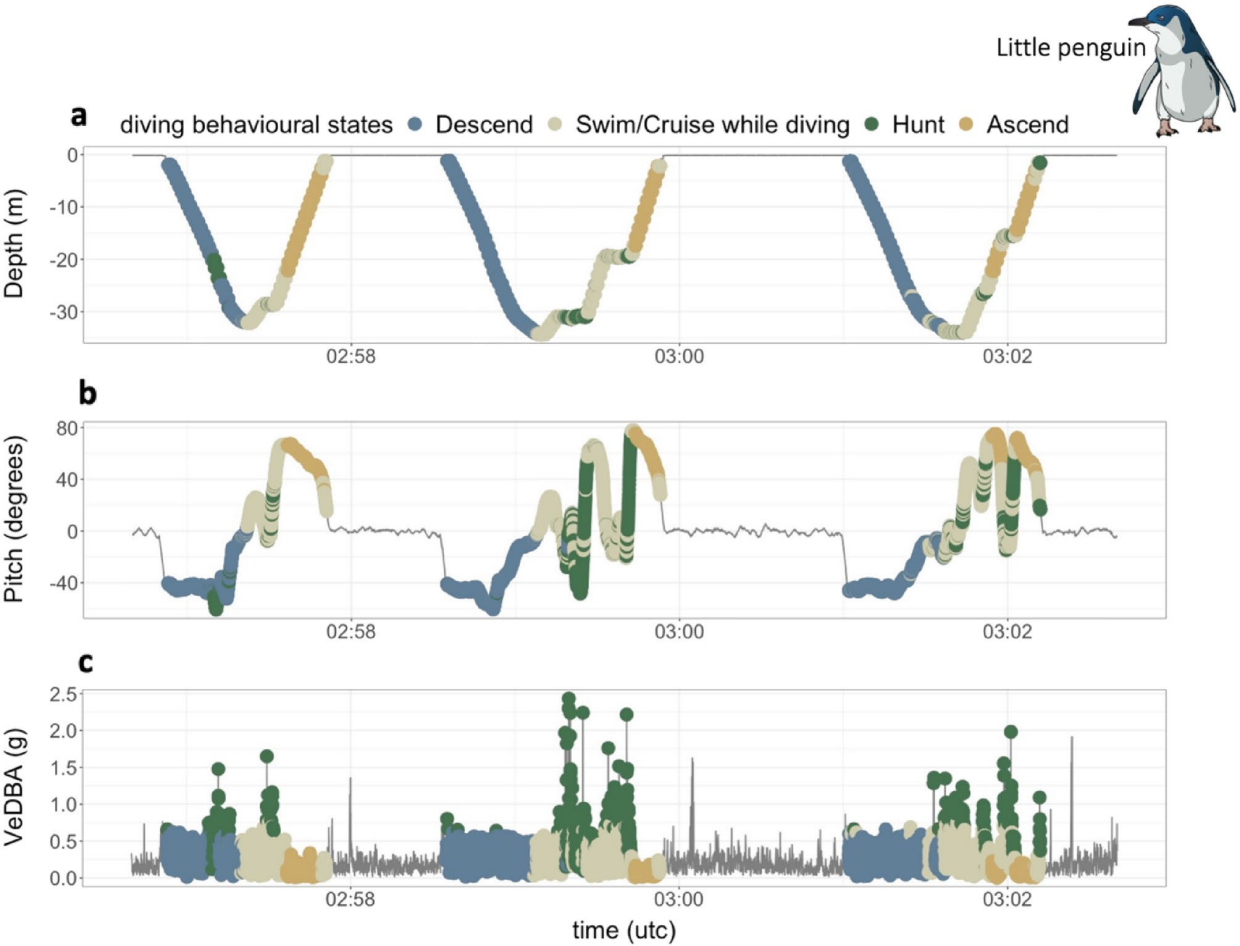
Example of behavioural classification in Little penguins (*Eudyptula minor*) while diving. (**a**) diving profiles, (**b**) Pitch (degrees) indicating body posture while diving, (**c**) Vectorial Dynamic Body Acceleration (VeDBA, g) indicating overall acceleration while moving underwater. Colours indicate the behavioural states identified while diving.

Ten behavioural classes were identified for little penguins. As for diving Adélie penguins, we identified “descend “, “ascend “, “hunt “ and “swim/cruise “ (Fig. 3). For *Season 2*, we additionally identified the swimming behaviour “swim/cruise type 2” in one foraging trip (out of 39). While at the sub-surface/air interface we identified “slow swim “ and “swim/porpoise “ while moving horizontally at the sub-surface and “preen/high flap on water “ behaviours plus one additional resting behaviour not found in Adélie penguins, termed “still” and characterized by the animals floating on the water surface without any detectable activity other than a change in posture (SI Appendix Fig. S5). For both species “swim/cruise type 2” was included in the “swim/cruise” general behaviour for the *Random Forest* (RF) models, because of its similar biological meaning to “swim/cruise” and because it was identified in very few individuals (see Methods for more details).

### Supervised machine learning approach

**Figure 4:**
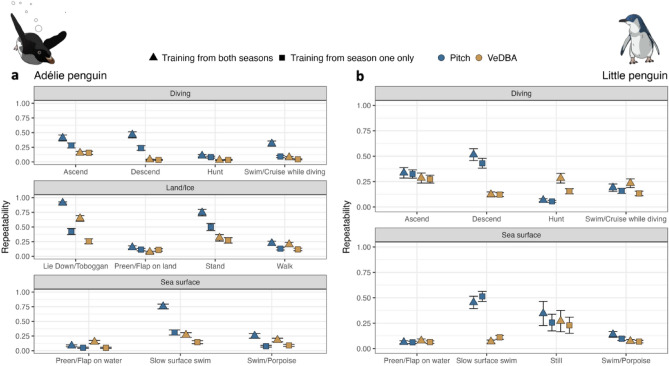
Repeatability scores. Values are calculated for pitch (blue) and VeDBA (yellow) on training datasets obtained from *Season 1* (squares) and both seasons (triangles) for both species, Adélie penguin (*Pygoscelis adeliae)* (**a**) and little penguin (*Eudyptula minor*) (**b**).

Repeatability scores for the training datasets from both species generally showed a lower inter- individuals’ variation in VeDBA than in pitch (Fig. 4). Including both sampling seasons within the training datasets resulted in higher variation in pitch for some behaviours and mainly for Adélie penguins, as “lie down/toboggan” and “stand” performed on land/ice, or “ascend” and “descend” (Fig. 4). The Out-Of-Bag (OOB) error estimated by the RF algorithm (measuring the prediction error of the model) showed a minimal decrease with increasing number of trees for both species (SI Appendix Table S2). Based on the minimal decrease, the model with 1000 trees was selected for both species to avoid any potential overfitting. Pitch, depth, change in depth, the standard deviation of raw heave at 2 s, the standard deviation of the roll at 30 s and the standard deviation of heave at 10 s were the six most important variables for Adélie penguins, with VeDBA taking the seventh position (SI Appendix Fig. S6). Depth, the standard deviation of the roll at 30 s, the standard deviation of heave at 2 s, the change in depth, VeDBA and pitch were the six most important variables for little penguins (SI Appendix Fig. S7). For Adélie penguins, behaviours such as “walk”, “swim/porpoise”, “slow surface swim” and “preen/flap on the water” were characterised by a higher degree of confusion compared to “ascend” and “descend”, for example. Hence observations originally labelled as “walk” could be confused as “stand” or “preen/flap on land” by the RF. The degree of confusion across behaviours was also confirmed with the Receiver Operating Characteristic curve (ROC), with curves closer to the top left corner indicating a better performance (SI Appendix Table S3 and Fig. S8). Similarly, for little penguins, behaviours such as “swim/cruise” while diving, “swim/porpoise” and “still” had a higher degree of confusion, and could have been labelled as other behaviours and displayed a lower ROC (SI Appendix Tables S4 and Fig. S9).

**Figure 5:**
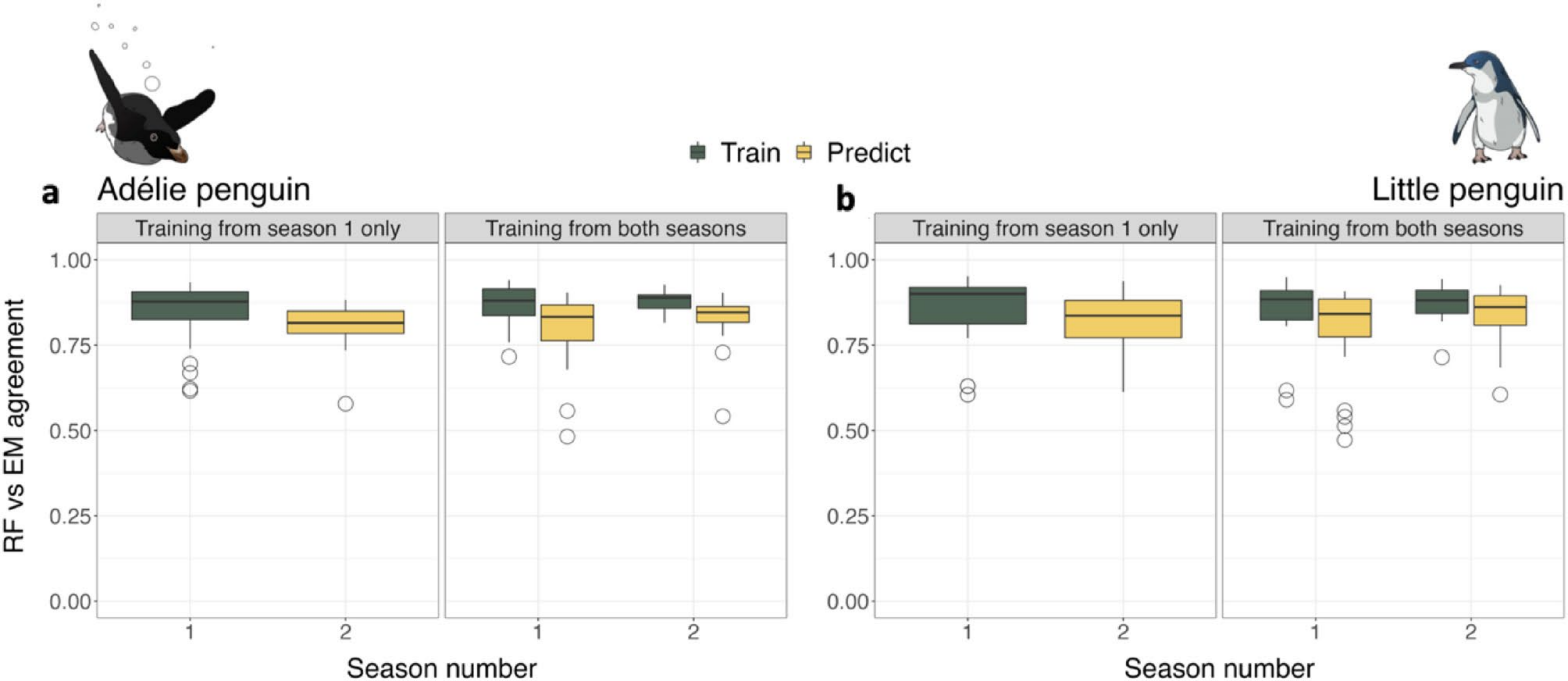
Agreement between machine learning approaches. Overall agreement between behavioural classification performed by the unsupervised machine learning algorithm *Expectation Maximisation* (EM) and one returned by the supervised machine learning algorithm *Random Forest* (RF), ran on data collected during foraging trips of Adélie penguins (*Pygoscelis adeliae)* (**a**), and little penguins (*Eudyptula minor*) (**b**).

The overall accuracy (hereafter agreement between the two methods, as proportion of identical behavioral prediction estimated by the two approaches within the given dataset) was high, above 0.8 (Fig. 5). However, a few outliers indicated low agreement on some individuals and a slight drop in the level of agreement was observed between the part of datasets used for training and the part used for predicting, so unknown to the RF. Specifically, for Adélie penguins, the agreements between the methods EM and RF were 0.85 ± 0.06 for the model trained on *Season 1* only, and 0.81 ± 0.05 for the prediction on *Season 2*. For the model trained using data from both seasons, the agreement levels were 0.87 ± 0.06 and 0.88 ± 0.02 for the part of the datasets respectively from *Season 1* and *Season 2* used for training; and 0.81 ± 0.07 and 0.83 ± 0.07 for the part of the datasets used for predicting (Fig. 5).

Similarly, for little penguins, the agreement between the methods were 0.86 ± 0.09 for the model trained on *Season 1* only and 0.81 ± 0.08 for the prediction on *Season 2*. For the model trained using data from both seasons, the agreement levels were 0.85 ± 0.09 and 0.87 ± 0.05 for the part of the datasets respectively from *Season 1* and *Season 2* used for training, and 0.80 ± 0.1 and 0.84 ± 0.08 for the part of the datasets used for predicting (Fig. 5).

### Effect of differences in behavioural classification on time-budgets and estimates of energy expenditure

**Figure 6:**
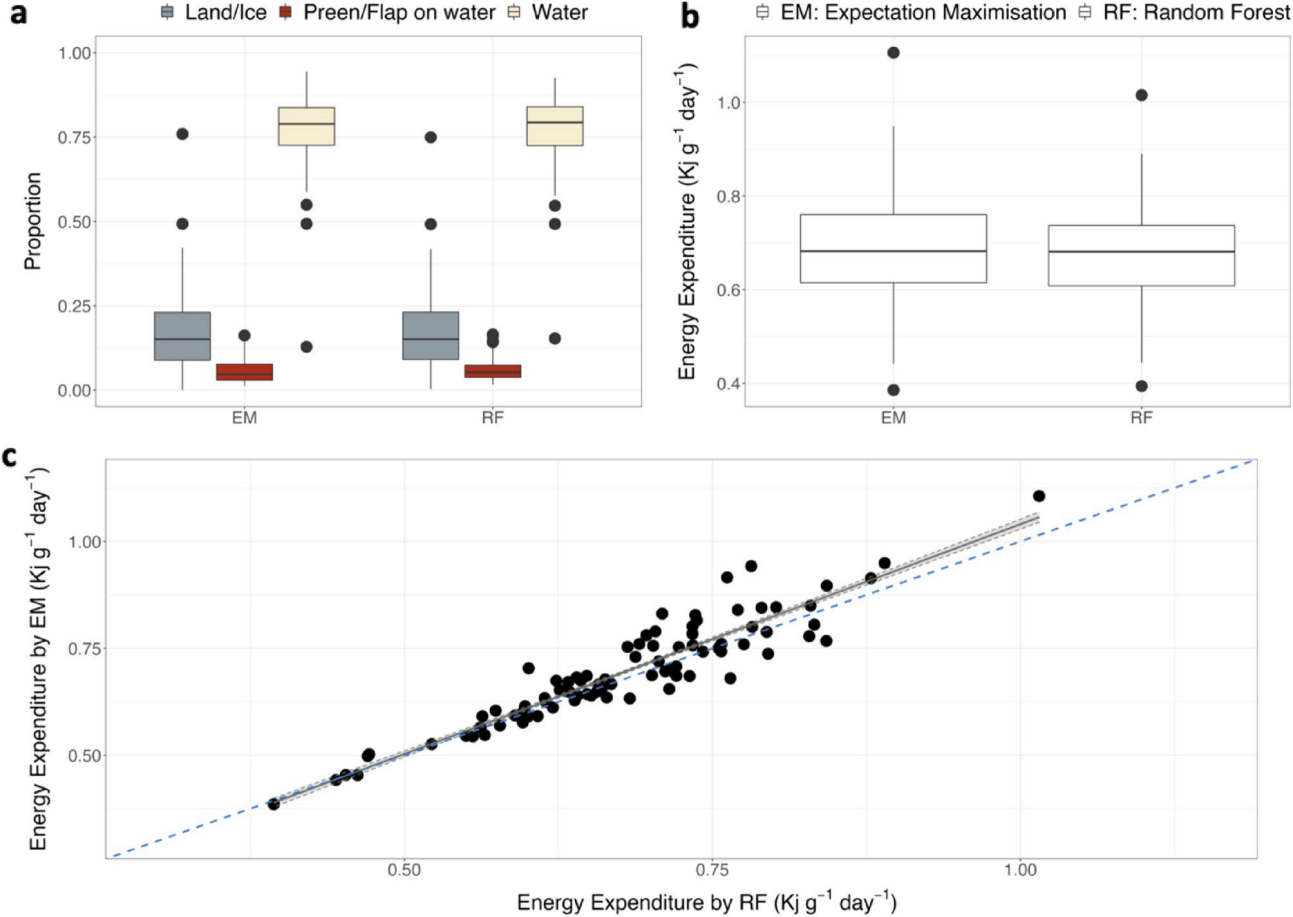
Daily Energy Expenditure estimated using results from two machine learning approaches. Energy Expenditure obtained accounting for the activity budgets predicted by unsupervised machine learning algorithm *Expectation Maximisation* (EM) and the supervised machine learning algorithm *Random Forest* (RF) for Adélie penguin (*Pygoscelis adeliae).* (**a**) activity budgets used for calculating energy expenditure as in Hicks et al.*2020*, (**b**) boxplots of the distributions of the resulting Energy Expenditure, (**c**) comparison and regression between the Energy Expenditure calculated with the two approaches.

As predicted by the confusion matrices and overall agreements, the differences in behavioural classification by the two machine learning approaches, albeit small, gave rise to differences in fine-scale activity budgets (SI Appendix Figs. S10–S15). For both species, depending on the type of training used (from *Season 1* only or both seasons), some predicted budgets as “slow surface swim”, “still” and “stand” could vary more than others like “lie down/toboggan”, “ascend” and “descend” (SI Appendix Figs. S10–S15). For Adélie penguin, energy calculations were possible for 89 trips (38 individuals performing 1 to 3 trips each) as sex was not recorded for some individuals. Trip duration was 24.2 ± 6.6 h. Resulting behavioural classification was grouped in the three coarse behavioural components used in Hicks et al.^32^, “Preen/Flap on water”, “Water” and “Land/Ice”, showing that the overall proportions of time spent within the components and resulting DEEs estimated with the two methods were similar (Fig. 6). However, both differences in proportions of time spent in the water and preening on the water surface affected the relationship between estimates by the two methods (*p* value < 0.001, Fig. 6 and SI Appendix Table S5). The model with a difference in the proportion of time spent preening on the water as covariate had the lowest AIC (adjusted R-squared: 0.98, Table S5). It showed that, while DEE estimates by the two methods are similar for the same individual, the DEE estimated by the EM method could be slightly higher than the one estimated by the RF method (Fig. 6). As consequence, total energy expended per trip followed the same trend, being lower when estimated by the RF compared to the EM (Table S5).

## Discussion

Our study revealed that behavioural classification obtained from big data recorded with accelerometers can be transferred across individuals within the same population and upscaled. Using a large sample size and including behavioural variability within training datasets resulted in a high level of accuracy (> 80%) between the supervised and unsupervised approaches. However, a decrease in accuracy is expected when predicting on unknown data using the supervised machine learning approach *Random Forest*. The low predictive performance has been suggested to lie within individual intraspecific differences^47,48^. Our reduced accuracy of 3–6%, between the models with different training datasets, can be associated to reduced sample size used in the model including individuals from both seasons. This drop, is, however, small compared to previously published values (30–35%), resulting from our overall larger sample size^48^. Few individuals with low predictive performance (around 50% of agreement between approaches) could be identified in our case, highlighting that some behaviours were classified differently by the two methods. Hence, individual variation was not completely accounted for within the training sets. Lower predictive performance could be associated, for example, to differences in diving angles during the descent phase, leading one algorithm to associate this posture to a “swim/cruise” behaviour rather than “descend” as in the other. We advise the broad scientific community approaching the large datasets collected with accelerometer data to be cautious when I) using training datasets obtained from few individuals and II) predicting behaviours over large numbers of individuals. Comparison between unsupervised and supervised approaches can help detect individual variability within the individuals sampled. Increasing number of individuals, data points randomly assigned to the training datasets, as well as number of variables could improve model accuracy but at the cost of making models increasingly slow and memory hungry. Implementing feedback mechanisms and further parallelisation of machine learning tasks would be a step towards more efficient management of these increasingly large datasets.

One of the main aims in ecology is to understand the behavior and physiology of, and environmental conditions experienced by, animals as they move through and interact with their environment. The use of bio-logging technology has allowed researchers to observe and quantify behaviours of species difficult to monitor otherwise. It is, however, difficult to obtain behavioural validation on species’ behaviours and variations. Although camera recorders are beginning to be widely used to validate behaviours, they usually collect data for short periods of time and on a few individuals, still not capturing inter-individual variability. Moreover, for the specific case of underwater images, data are affected by light and water turbidity and are still difficult to process automatically^49^. The use of deep learning techniques is making it easier and faster to extract information from images^50^. The variables used in our models have been validated for predicting hunting behaviour in similar species^51^.

Signal overlap across similar behaviours (such as subsurface swimming behaviours in our case) and class imbalance (as some behaviours might be under-represented within the training datasets), might confuse^28,52^ and lead to disagreement between approaches used. Information on behaviours collected from deployments using camera loggers combined with accelerometer tags could help clarify and deal with confusion between some behavioural classes. Penguins are elusive species, moving in different habitats (land, ice, underwater) and dynamic environmental conditions. Furthermore, the two species considered in this study are marine predators foraging on lower trophic levels (e.g. feeding on fish and krill). Their behavioural variation as a result of encountered environmental conditions (e.g. prey availability) makes them sentinels of climate and ecosystem changes^53^ emphasizing the need to monitor their behaviour and understand how individuals modify it. Adélie penguins showed more inter-variability in behaviour than little penguins. However, for both species, predictions from models trained on data from both seasons showed similar agreement scores to the predictions made by models trained on data from a single season. Species, types of behaviour performed, and environmental conditions can shape the degree of variability recorded^54–58^. Changes in sea-ice conditions (as in extent and characteristics, for example), might lead Adélie penguins to move differently^59^ and perhaps show high variability in behaviour. On a broader perspective, body conditions, age and changes in prey type and pursuing tactics, for example, can lead to changes in underwater movements, thereby affecting individual fitness (survival and reproduction)^59–61^. By implementing machine learning approaches able to analyse the vast amount of data collected with bio-logging devices recording high-resolution movements, it would be possible to start assessing the effect of environmental variability on individuals’ behaviours and energetics.

In bio-logging and movement ecology, analyses on behavioural variation linked to environmental conditions are carried out primarily on location data, which currently constitute the most extensive animal movement dataset in terms of the number of individuals and species tracked^55^. Accelerometer datasets are also becoming suitable for these analyses^62^. However, differences in tag attachments across multiple sampling seasons (due to different researchers attaching the loggers, to double tagging experiments or tag positions) can cause discrepancies in accelerometer-derived variables, resulting in variability in the recorded behaviour^12,63,64^. We used pitch and VeDBA as candidates for body position and body motion. We accounted for possible differences in tag attachment by adjusting pitch values considering the subsurface slow swim/swim as the most common behaviour from which the tag “*null position*” could be identified. Standardizing accelerometer data across sampling seasons will help identify the effects of individual variability, and different environmental conditions on both animal behaviours and energy expenditure^64^.

Acceleration-based proxies for energy expenditure can provide new insights in optimal behavioural and evolutionary foraging theory, fitness and survival-related consequences of changes in energy expenditure^12,65–67^. It can potentially be used to explain how large-scale patterns at population levels may arise. An increasing amount of studies show that dynamic body acceleration, activity-specific dynamic body acceleration and activity budgets derived by accelerometer tags can predict energy expenditure, as measured via doubly labelled water in free-living animals^16,32,68,69^. Depending on the relationship found, as in number, type and resolution of behaviours estimated, the formulas linking accelerometer derived metrics with energy expenditure might be sensitive to different time budgets estimated via other methods. In our case, some of the fine-scale behavioural classes were grouped in coarser categories, leading to minimal differences in coarse time budgets and energy expenditure estimates. For Adélie penguins, our daily energy expenditure values predicted for *Season 1* fell within the range of values validated for *Season 2*, despite the use of slightly different behavioural classification methods^32^. Also the estimates for the total energy expended per trip fall within the range presented in Balance et al.^70^. However, this might not always be the case. We advise caution when using and mixing machine learning approaches to predict behaviours and energy expenditure, as this might cause variation in the resulting energy estimations.

Behavioural classification methods, used in ecology and bio-logging fields, based on a good understanding and careful examination of the acceleration signals can help improving computational time and accuracy of approaches used. Both unsupervised and supervised machine learning approaches applied to accelerometer data can provide insights into the behavioural ecology of wild animals from different perspectives but come with both benefits and costs. The unsupervised machine learning approach *Expectation Maximisation* allowed us to independently estimate behaviours for single individuals without using a priori knowledge of types of behaviour performed. Checking the behavioural classification of single foraging trips can be, however, time consuming and might lead to misinterpretation with increased behavioural complexity. The supervised machine learning approach, *Random Forest*, allowed us to upscale our analysis, build and test large training datasets across the two species sampling from the variability of behaviours available within our datasets. Random Forest, in fact, allows for quantification of the uncertainty around each estimated behaviour by means of confusion matrices. While computational speed was gained, it led to a potential loss of information on individual variability. These aspects are of vital importance when using behavioural classification’s model outputs for testing theories on animals’ fitness, energetics, and behavioural adaptation, as well as for informing conservation research.

Finally, training datasets accounting for behavioural variability could be applied to increasingly larger databases and/or used for real-time processing of accelerometer data on board of bio-logging devices to facilitate transmission via satellite systems^27^. While our framework has been tested on two penguin species, it is transferable to other species and systems. Methodological advances in computer science are allowing ecologists to handle and analyse large datasets more efficiently, but interpretation and transferability of results are tasks that need improvements. Refining the methodological applications calls for more integration between ecology and data science fields, along with standardizing the heterogeneous datasets collected, upscaling both data collection and analysis and interpretation of complex ecological patterns.

## Methods

### Ethics statement

Field work protocol for both species was approved by the Terres Australes et Antarctiques Francaises (TAAF), the French regional ethic committee 54 for the Antarctic-based studies, and the ethics committee of the Phillip Island Nature Park Animal Experimentation Committee with a research permit issued by the Department of Environment, Land, Water and Planning of Victoria, Australia. All experiments were performed in accordance with relevant guidelines and regulations.

### Data collection

Adélie penguins (*Pygoscelis adeliae*) were studied at the colony on Ile des Pétrels, at the Dumont d’Urville station, in Terre Adélie, East Antarctica (66°40′ S; 140°01′ E), over two breeding seasons, 2019–2020 (*Season 1, reference season*) and 2018–2019 (*Season 2, testing season*). The birds that we considered were breeders during the chick guarding stage, in which one parent guards the chicks on the nest while the other is foraging at sea to bring food back to its offspring. Only one member of a pair was captured on or when leaving the nest, when both adults were attending the nest before/during a changeover. Little penguins (*Eudyptula minor*) were studied at Penguin Parade® on Phillip Island, Victoria, Australia (38° 31′ S, 145° 09′ E). Birds considered were breeding in artificial nest boxes and were sampled across incubation, guard and post-guard stages over two breeding seasons, 2020–2021 (*Season 1, reference season*) and 2019–2020 (*Season 2, testing season*). For both species, data-loggers (Axy-Trek, Technosmart, Italy, 40 × 20 × 8 mm, 14 g) were attached to the central dorsal region of the bird^71^. The loggers continuously sampled tri-axial acceleration at 100 Hz for Adélie penguins and 25 Hz for Little penguins, as well as pressure (in millibars) at 1 Hz and GPS locations (for Adélie penguins: 60 s; for little penguins in 2019–2020 300 s during incubation, 10 s during guard and 60 s during post guard, and in 2020–2021 30, 60 and 300 s during incubation, 10 s during guard and 60 s during post guard). GPS locations were only used during data preparation.

### Data preparation

Acceleration data were checked and subsampled at 25 Hz, to be able to compare the two species across the analysis. Raw acceleration data are recorded over three axes: surge (back-forward acceleration), sway (lateral acceleration) and heave (dorso-ventral acceleration). Using a 2 s running mean over the raw acceleration signals for both species, we first calculated static acceleration, providing a measure of the angle of the instrumented animals. The time window applied for the running mean was arbitrarily chosen based on Shepard et al.^72^ as a compromise to reduce noise and avoid masking behaviourally driven signals. By subtracting the static acceleration values from the raw values, we calculated dynamic acceleration for the three axes, measuring the change in velocity due to animal body motion. From the static and dynamic acceleration, vertical body orientation (pitch), lateral body orientation (roll), and Vectorial Dynamic Body Acceleration (VeDBA) were then calculated, following Hicks et al.^32^; but see also R code. It was assumed that different behaviours could be performed at different temporal resolutions^20^. Hence the following additional variables were also calculated: standard deviation of the raw acceleration values from the three axes over a running mean of 2, 10, 30 and 60 s, standard deviation of roll over 30 s and standard deviation of pitch and VeDBA over 60 s.

Pressure in millibars was converted to water depth (m) by subtracting the mode of the pressure distribution from the raw pressure and dividing by hundred. According to the resolution of the loggers and waves at the surface, only dives > 1 m and > 2 m, respectively, for Little and Adélie penguins, were considered diving behaviour^32,73^. Depth change rate was also calculated. Single versus multiple foraging trips were then defined by looking at GPS locations, depth, pitch, and temperature channels to detect when the animals were first entering the sea and when they were back at the colony. For Adélie penguins, we obtained 107 foraging trips (47 individuals) for *Season 1* and 53 foraging trips (48 individuals) for *Season 2*. For little penguins, we obtained 59 foraging trips (53 individuals) for *Season 1* and 39 foraging trips (38 individuals) for *Season 2*. Data analysis was performed in R version 4.0.4^74^ on a processor of 3 GHz, 10-Cores and 128 GB of RAM. Distributions are presented as mean ± sd.

### Unsupervised machine learning approach

Both species can perform several behaviours while moving in the wild. Specifically: “walk”, “stand”, “lie down”, “toboganning” while on land (ice for Adélie penguins only), slow swim, preen, swim or porpoising at the subsurface level and ultimately descend, ascend, hunt and swim/cruise while diving^75^. To be able to detect these behaviours without a priori knowledge available, we followed the approach implemented in^20^, which used the unsupervised machine learning algorithm “Expectation Maximisation”, (hereafter EM). The algorithm is implemented in the R package “*Rmixmod*”^76^ performing clustering analysis fitting a mixture model of multivariate Gaussian or multinomial components to given data. The algorithm selects an initial setting for the parameters, denoted *h*_i_. Given a joint distribution over observed variables X and latent variables Z, governed by the chosen parameters, the algorithm maximises the likelihood function. The values of the latent variables in Z are given by the posterior distribution considering the expected value of the log-likelihood under the posterior distribution of the latent variable. In the Mstep, the algorithm evaluates *h*_new_, checking for convergence of either the log-likelihood or the parameter values. If the convergence criterion is not satisfied, the algorithm returns to select the initial settings for the parameters recalculating *h*_i_^20,77^.

We have simplified the analytical procedure given the animals move in different dimensions (horizontal, as space, and vertical, as depth) and environments (land/ice and water). Foraging trips were split into coarse subsets representing when the animals were on ice (for Adélie only), at the water surface and diving, according to rules considering pitch (i.e. > 60 degrees to detect a penguin walking), and/or temperature (generally > 0ºC ºC when walking on ice) and depth as mentioned above (see R code and^32^). The subsets were then visually checked for potential errors. For each subset (2 or 3 per foraging trip, whether ice/land events were detected or not), variables were scaled and centered and the EM was randomly initialised by setting “*random*” within the “*mixmodStrategy”* function. Initialisation from a random position is a standard way to initialise the EM algorithm. This random initial position is obtained by choosing at random centres in the dataset. Based on the knowledge of the behavioural ecology of the species, EM runs for the diving subset were set to detect a minimum of three behavioural clusters and a maximum of eight. EM runs for the subsurface and ice subsets were set to detect two to five behavioural clusters. If only subsurface or ice events were detected, EM runs were set to run to detect from two to eight behavioural clusters. Variables fed in the algorithm were selected following an iterative approach consisting of considering the general knowledge of the environments where the data were collected, the general behaviours known about the study species (walk, preen and dive) and the statistical properties of the variables calculated^20^, but see also *Results* section. Foraging trips for each individual and season were run separately, and all behavioural clustering combinations recorded. Resulting clusters from each foraging trip analysed with the EM algorithm were visually assessed and associated to a behavioural class summarising the general behaviour performed (i.e., “descend”, “hunt”, “walk”).

### Supervised machine learning approach

A set number of observations for each behavioural cluster was randomly drawn from each foraging trip to prepare the training dataset for the supervised machine learning approach. Only the Little penguins’ dataset had recordings across different breeding stages. Breeding is not synchronized in this species and dive parameters overlap between breeding stages^78^, hence each foraging trip had the same probability to be sampled. The number of sampled observations was selected as the minimum between half of the observations from the smallest behavioural cluster and 1000. This way, it was possible to always leave at least half of the samples of each behavioural cluster for the test set and use at most half of the samples for training. The 1000 value is arbitrary and is only used to ensure that the number of samples taken from each trip is not diverging too much (i.e., each trip gets to contribute to the classifier with similar amount of data). To compare individual trips and seasons and account for possible differences in tag positioning, pitch values were adjusted by subtracting the median value of the Pitch considered as near the surface (values equal and above 2 m and less than 60 degrees for Adélie penguins, and values equal and above 1 m for little penguins). As the behavioural cluster “Swim/Cruise type 2” (see Results section) was performed by a few individuals for both species resulting in too few observations within the training, it was included in the general “Swim/Cruise” behavioural class.

To test the level of individual variation contained in the training datasets, we ran a repeatability analysis on both datasets using the R package “*rptR*”^79^. We used pitch and VeDBA as candidate variables to assess the repeatability on the overall body orientation and acceleration for each behaviour. Both pitch and VeDBA were used as response variable in two separate models, the reference “group” was set as individual bird with a set of consecutive trips. The repeatability index ranges from 0 to 1: a low repeatability (near 0) reflects either a high intra-individual variation or a low inter-individual variation.

We used the machine learning algorithm “*Random Forest*”, (hereafter RF), implemented within the R packages “*tidymodels*” and “*ranger*”^80,81^. The RF algorithm was trained over twelve variables (see Table S1 and Results section) and parameterised on different numbers of trees (specifically 100, 500, 1000 and 1500). The parameter “*mtry*” indicating the number of variables randomly sampled as candidates at each split, around the sqrt(n max variables), was set to = 4. The randomly sampled training dataset obtained from all trips was split into further training (75%) and testing (25%) and a tenfold cross validation procedure performed on the final training data. Model selection was done by looking at the Out-Of-Bag error (OOB) and the Receiver Operating Characteristic curve (ROC) showing the performance of the classification model and the Area under the ROC Curve (AUC, see R code). The ROC curve shows the trade-off between sensitivity (or TPR) and specificity (1 – FPR).

### Comparing the classification approaches across sampling seasons

We set up two sets of models to answer our research questions I and II (Fig. 1). The first model, containing random samples from each trip within the same season (*Season 1*), was built and validated on *Season 1* and used to predict on the second season (*Season 2*). The second model contained random samples from trips from both seasons (*Season 1* and *Season 2*). As we collected different foraging trips per season, the number of trips to be randomly sampled was selected as half of the total number of trips from the smallest season in order to account for differences in sampling, limit the size of the final training dataset and leave trips for prediction. For example, *Season 1* for Adélie penguins accounted for 107 trips while *Season 2* had 53 trips. Hence the number of trips from which observations were sampled from each season were 26. The resulting model was used to predict the behavioural classes on the remaining trips from both seasons. This procedure was run for each species separately.

### Effect of using different behavioural classification approaches on time-budgets and energy expenditure estimates

Time budgets resulting from both EM and RF methods were calculated and compared. To answer question III (Fig. 1), we used the existing DEE vs activity-specific VeDBA relationship built and validated for Adélie penguins using data included in *Season 2*^32^. The model developed accounted for both VeDBA signatures, as well as time budgets. Thus, it was the ideal tool to fulfil our objective of investigating the consequences of using different behavioural classification methods. For the analysis, we only considered Adélie penguin data collected in *Season 1*. The RF algorithm used here, was trained solely on data from *Season 1*. We calculated DEEs for each trip using time budgets based on behavioural classes predicted by the EM and the RF, as well as total energy expended per trip, to be able to compare with other published estimates^70^. Following the best DEE model^32^, we also aggregated resulting behavioural classes in three coarse components: “Preen/Flap on water”, “Dive” (including sub-surface swimming behaviours, as well as behaviours within dives) and “Land/Ice”. We included the error estimates published in^32^ and individual sex which was required for the calculation and assigned following morphometric measurements. We used linear models to test the agreement between the DEE calculations from the two methods. We also tested different models by considering the difference in estimated coarse components as covariates. We checked for collinearity among explanatory variables, as well as model residuals. Model selection was done using Akaike's Information Criterion (AIC).

## Supplementary Information

Supplementary Information.

## Data Availability

Datasets used for this manuscript are available at: 10.57745/IT6ITA. Please cite as "CHIMIENTI, Marianna; KATO, Akiko; CHIARADIA, Andre; ROPERT COUDERT, Yan, 2022, "Data for - MuFFIN - Modelling Foraging Fitness in Marine Predators", 10.57745/IT6ITA, Recherche Data Gouv, V1". Sample dataset and all R codes used to manipulate and analyze the data used are available at https://github.com/MariannaChimi/MuFFIN_MSCA/releases/tag/v.02.Submission.
