# Supplementary material for: The role of individual variability on the predictive performance of machine learning applied to large bio-logging datasets
Source: Sci Rep. 2022 Nov 17;12:19737. doi: 10.1038/s41598-022-22258-1 (PMC9672113; doi:10.1038/s41598-022-22258-1)
Supplement: Supplementary file 1 — Supplementary Information. [file 41598_2022_22258_MOESM1_ESM.pdf]

Supplementary Information for

The role of individual variability on the predictive performance of machine learning applied to large bio-logging datasets.

Marianna Chimienti*, Akiko Kato, Olivia Hicks, Frédéric Angelier, Michaël Beaulieu, Jazel Ouled-Cheikh Bonan, Coline Marciau, Thierry Raclot, Meagan Tucker, Danuta Maria Wisniewska, Andre Chiaradia, Yan Ropert-Coudert

*Corresponding author. marianna.chimienti@cebc.cnrs.fr

This PDF file includes:

Figs. S1 to S17
Tables S1 to S5
References (1)

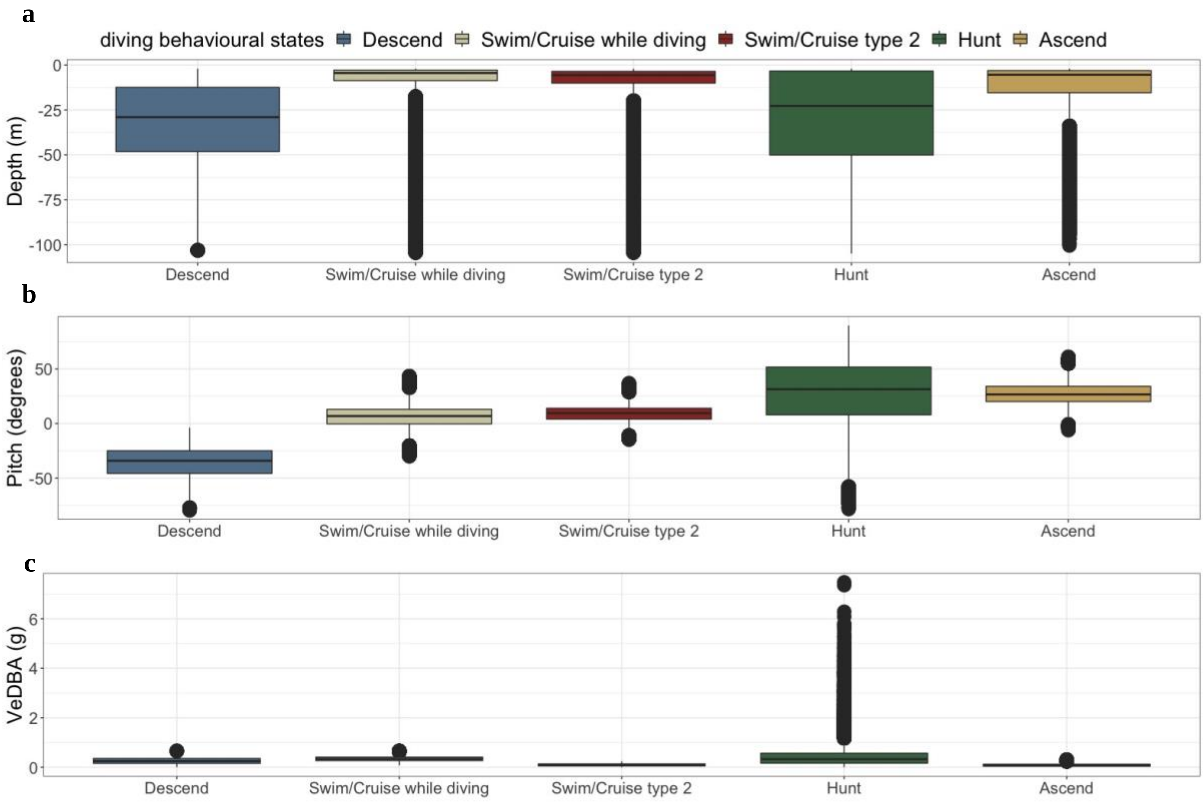

Fig. S1: Diving behaviours detected using the unsupervised machine learning algorithm *Expectation Maximization* on Adélie penguin (*Pygoscelis adeliae*). Example of distributions for depth (m) (a), pitch (body posture in degrees) (b) and Vectorial Dynamic Body Acceleration (body motion in g) (c) for the diving behaviours characterized using the unsupervised machine learning algorithm *Expectation Maximization* on Adélie penguin (*Pygoscelis adeliae).*

a

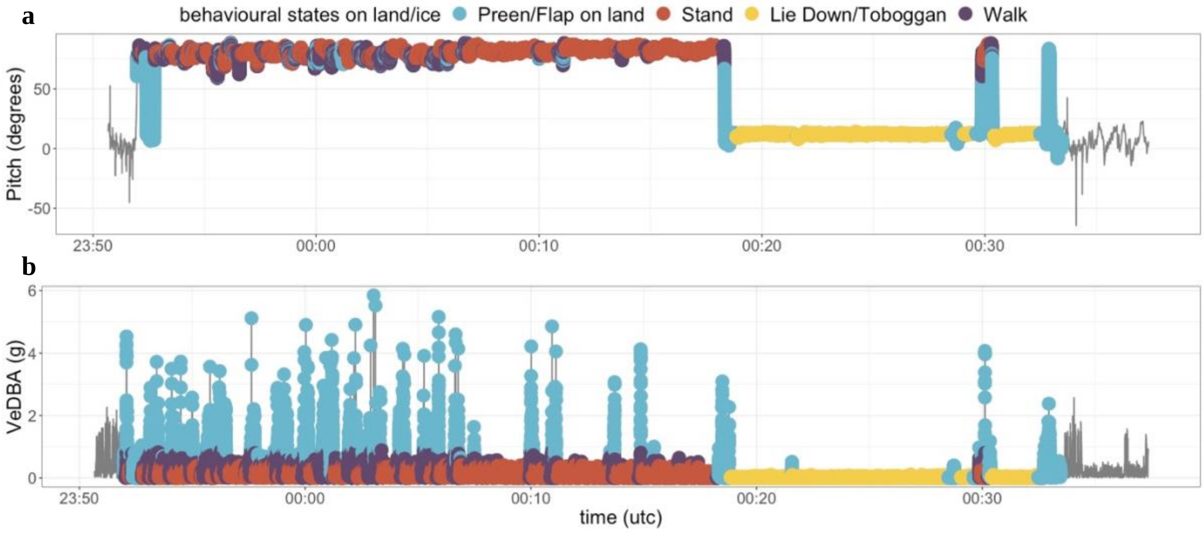

Figure S2: Land/ice behaviours detected using the unsupervised machine learning algorithm *Expectation Maximization* on Adélie penguin (*Pygoscelis adeliae*). Example of behavioural classification on pitch (body posture in degrees) (a) and Vectorial Dynamic Body Acceleration (body motion in g) (b) for the land/ice behaviours in Adélie penguin (*Pygoscelis adeliae*).

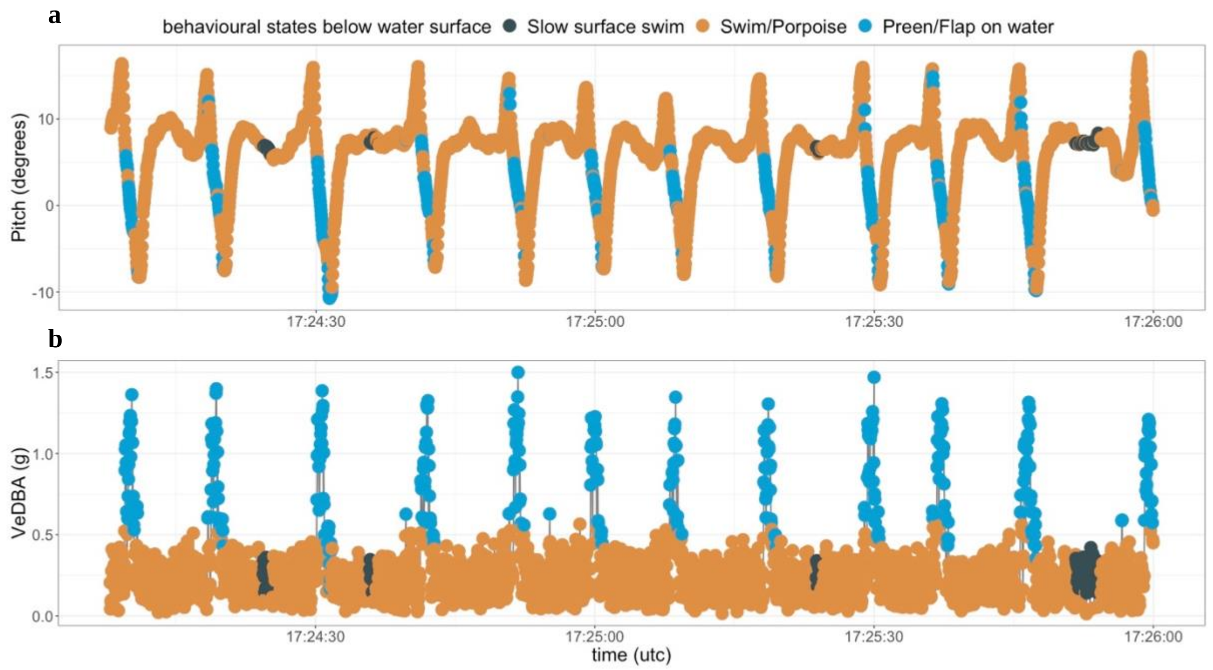

Fig. S3: Subsurface behaviours detected using the unsupervised machine learning algorithm *Expectation Maximization* on Adélie penguin (*Pygoscelis adeliae*). Example of behavioural classification on pitch (body posture in degrees) (a) and Vectorial Dynamic Body Acceleration (body motion in g) (b) for the subsurface behaviours in Adélie penguin (*Pygoscelis adeliae*).

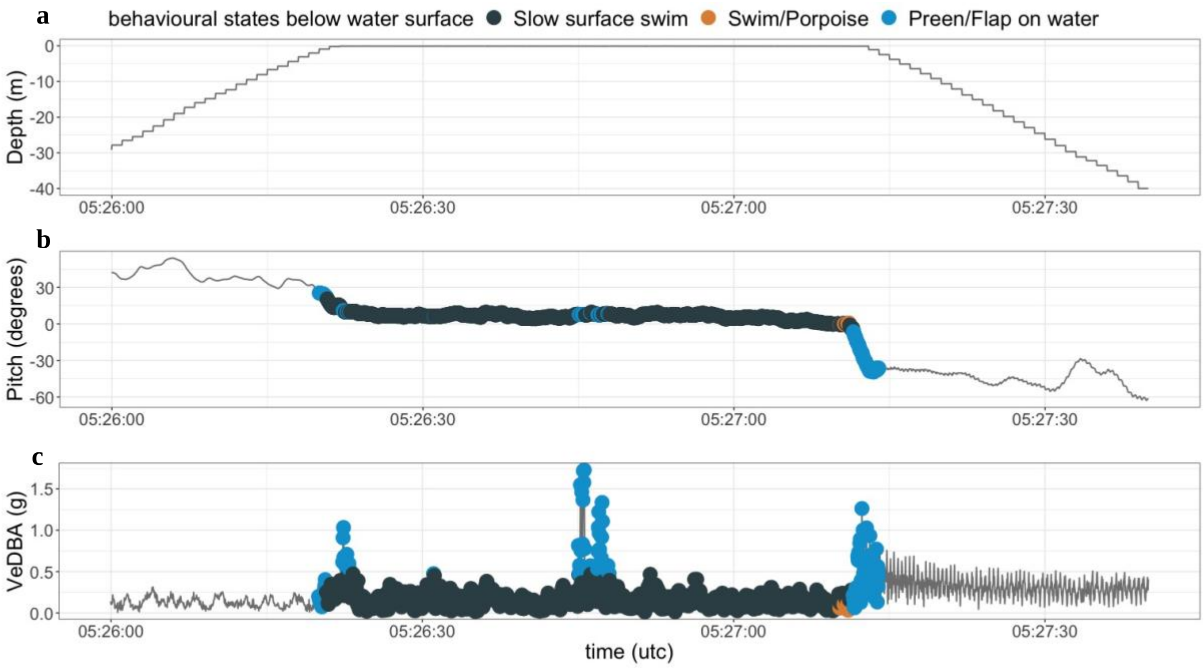

Fig. S4: Subsurface detected using the unsupervised machine learning algorithm *Expectation Maximization* on Adélie penguin (*Pygoscelis adeliae*). Example of behavioural classification showing mainly slow surface swim (depth= 0, a) on pitch (body posture in degrees) (b) and Vectorial Dynamic Body Acceleration (body motion in g) (c) for the subsurface behaviours in Adélie penguin.

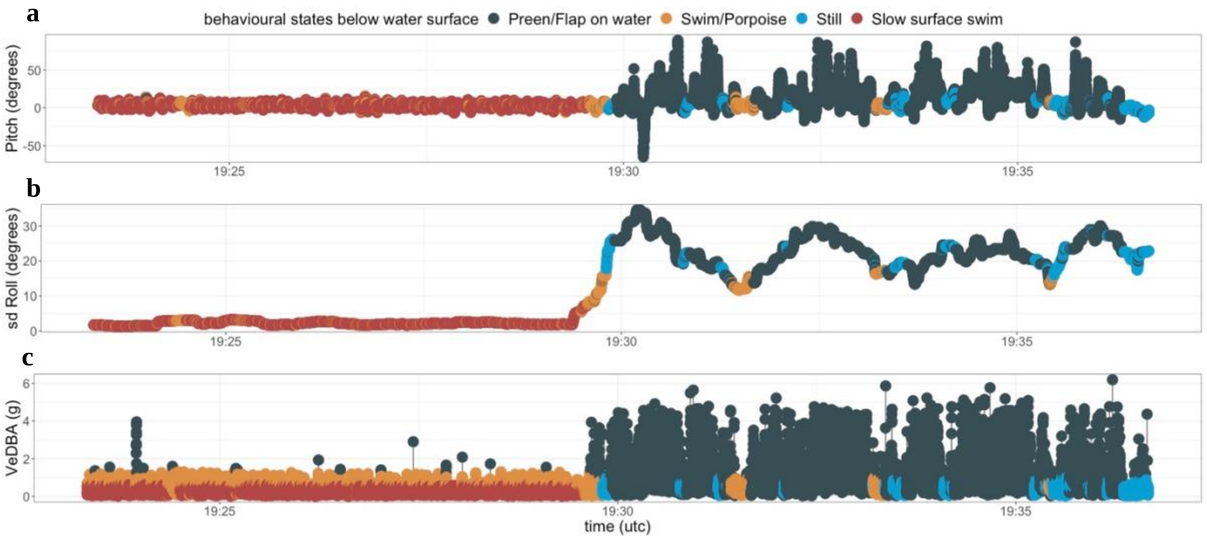

Fig. S5: Subsurface detected using the unsupervised machine learning algorithm *Expectation Maximization* on Little penguin (*Eudyptula minor*). Example of behavioural classification on pitch (body posture in degrees) (a), the standard deviation of roll (lateral body posture in degrees) (b) and Vectorial Dynamic Body Acceleration (body motion in g) (c) for the subsurface behaviours in Little penguin.

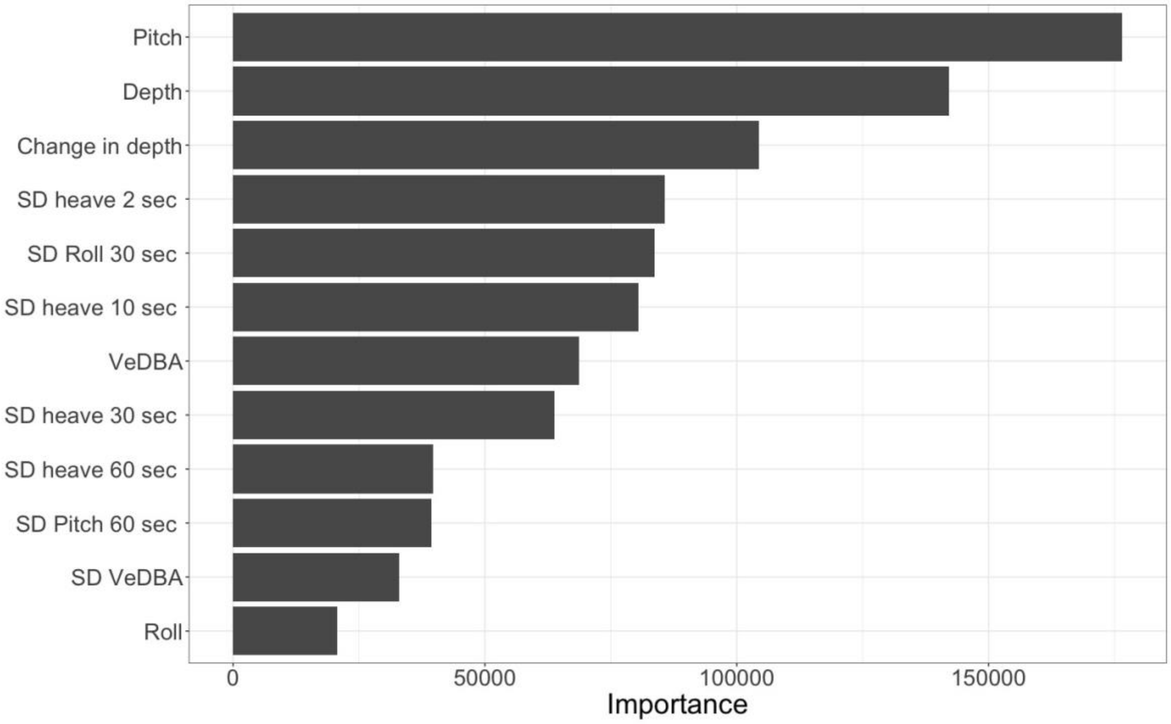

Fig. S6: **Variable importance obtained from Random Forest on Adélie penguin (*Pygoscelis adeliae)* indicating how informative each variable is for the model**. The model run with 1000 trees using training dataset obtained from *Season 1*.

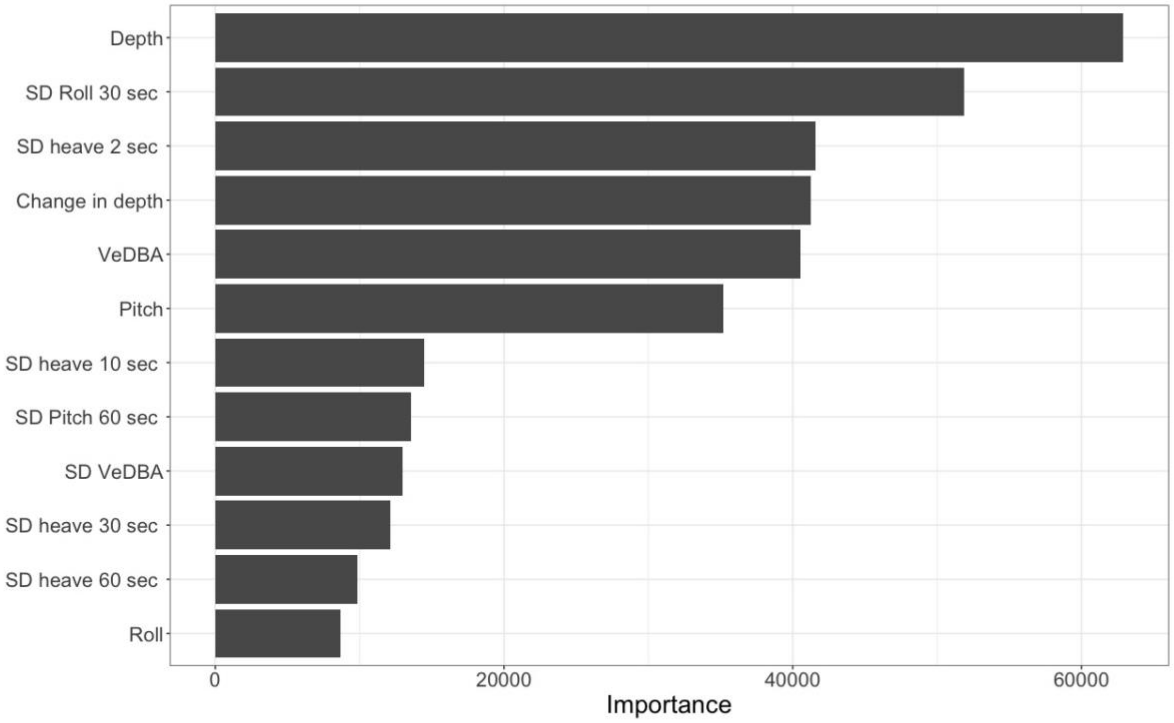

Fig. S7: **Variable importance obtained from Random Forest on Little penguin (*Eudyptula minor*) indicating how informative each variable is for the model.** The model run with 1000 trees using training dataset obtained from *Season 1*.

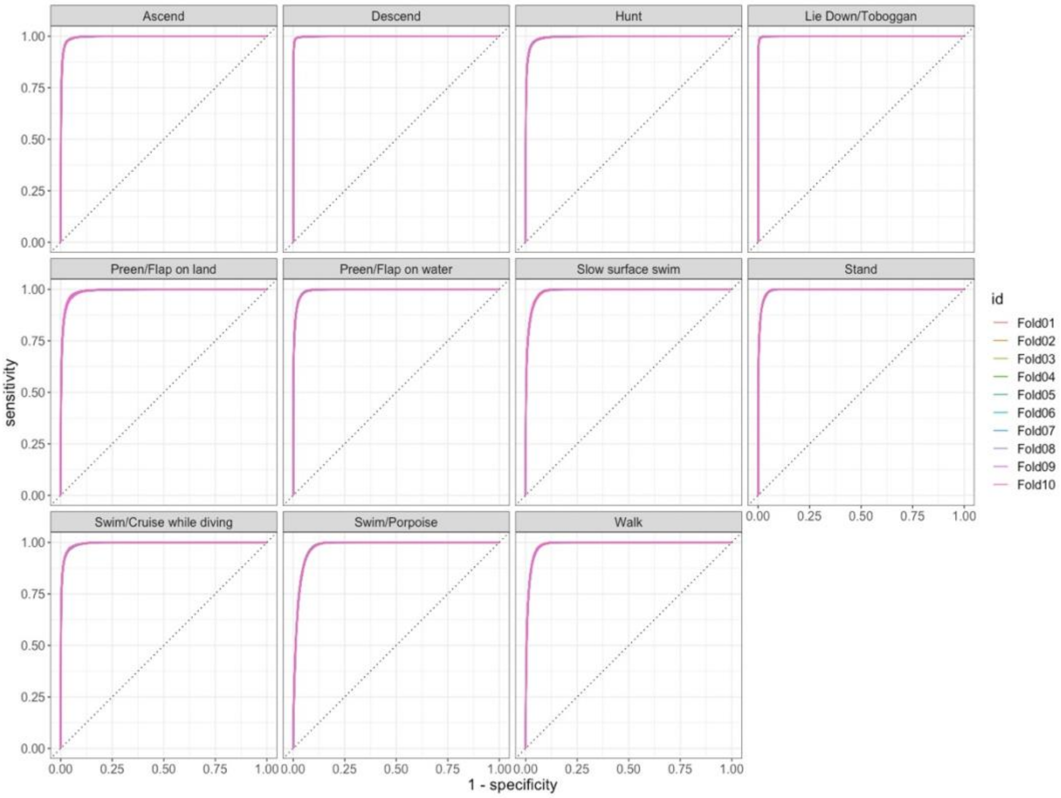

Fig. S8: **ROC curve obtained from Random Forest on Adélie penguin (*Pygoscelis adeliae)***. The model run with 1000 trees using training dataset obtained from *Season 1*.

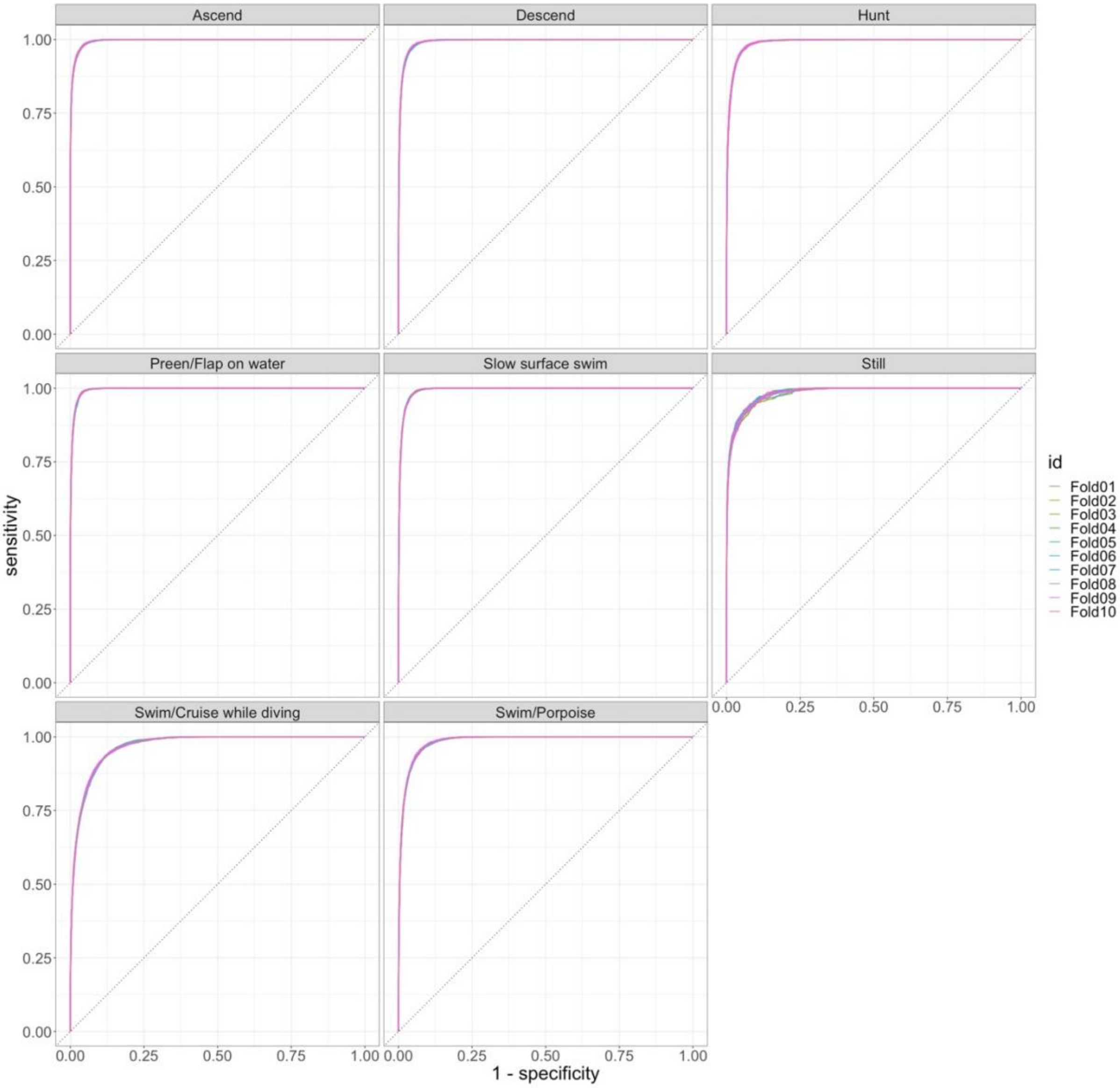

Figure S9: **ROC curve obtained from Random Forest on Little penguin (*Eudyptula minor*).** The model run with 1000 trees using training dataset obtained from *Season 1*.

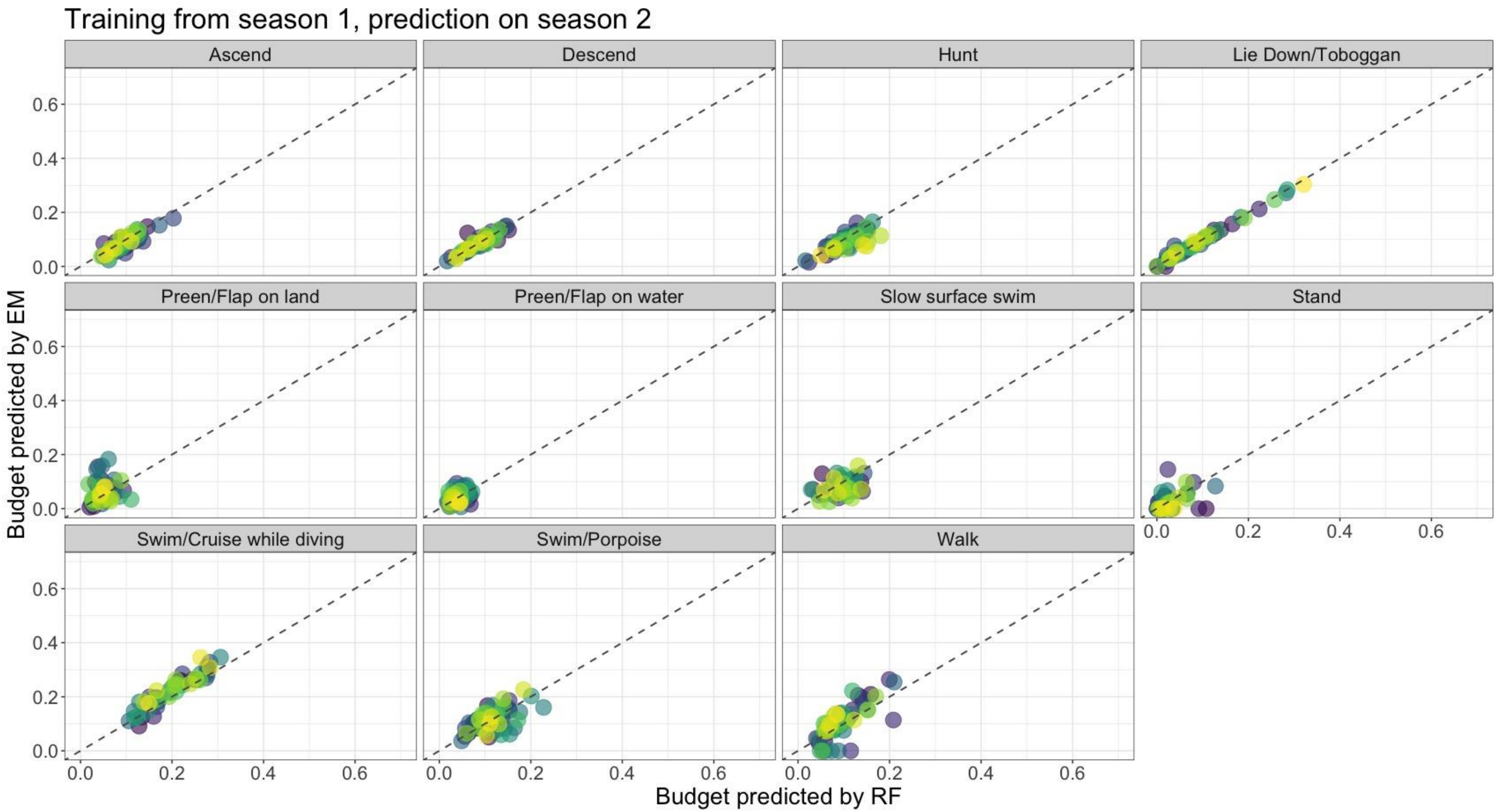

Fig. S10: **Comparison between activity budgets predicted on *Season 2* by the unsupervised machine learning algorithm *Expectation Maximisation* (EM) and the supervised machine learning algorithm *Random Forest* (RF) on Adélie penguin (*Pygoscelis adeliae*).** Budgets are calculated from the model runs considering training from *Season 1* only. Colours indicate individual foraging trips, dashed line a theoretical 1:1 relationship.

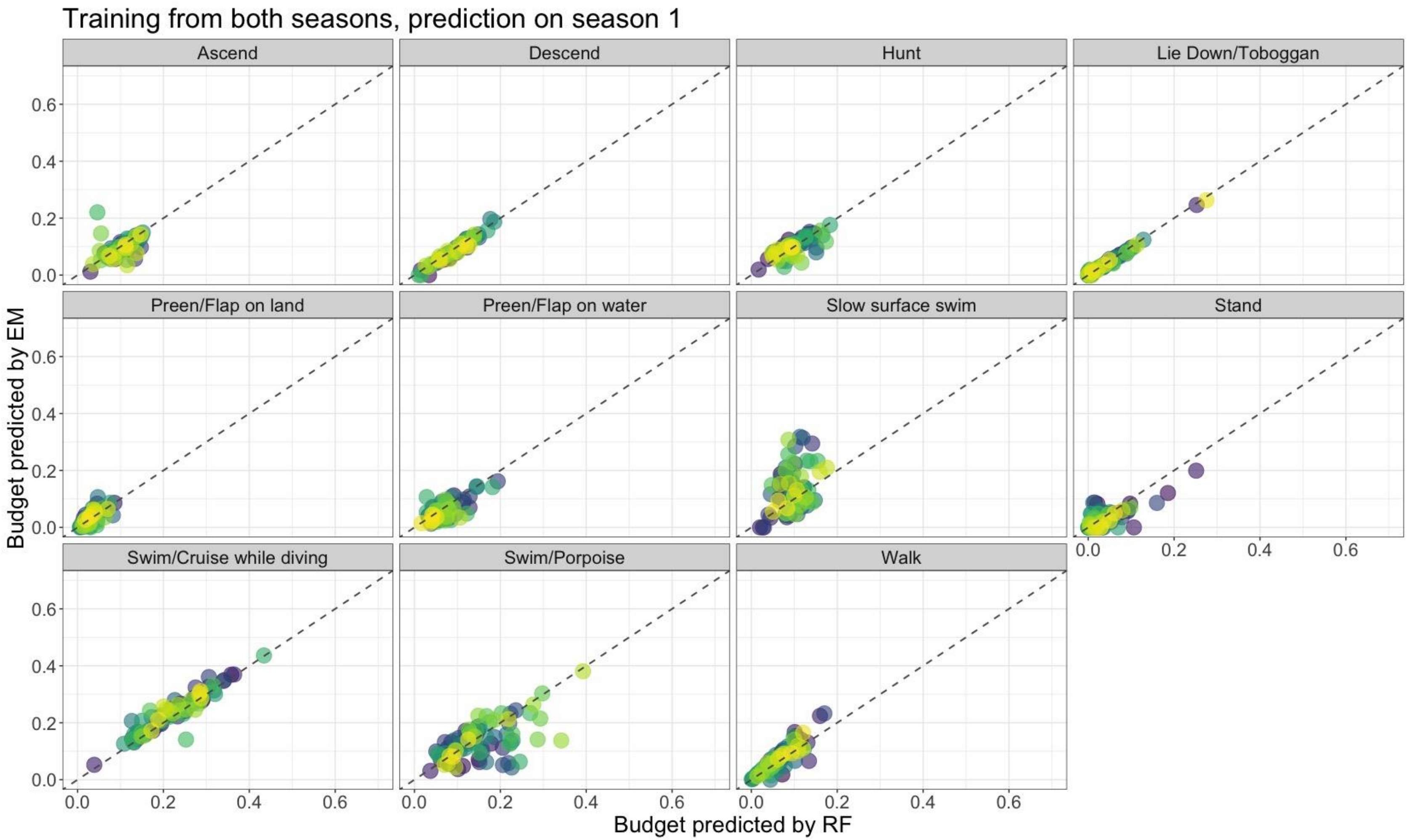

Fig. S11: Comparison between activity budgets predicted on *Season 1* by the unsupervised machine learning algorithm *Expectation Maximisation* (EM) and the supervised machine learning algorithm *Random Forest* (RF) on Adélie penguin (*Pygoscelis adeliae*). Budgets are calculated from the model runs considering training from *both seasons*. Colours indicate individual foraging trips, dashed line a theoretical 1:1 relationship.

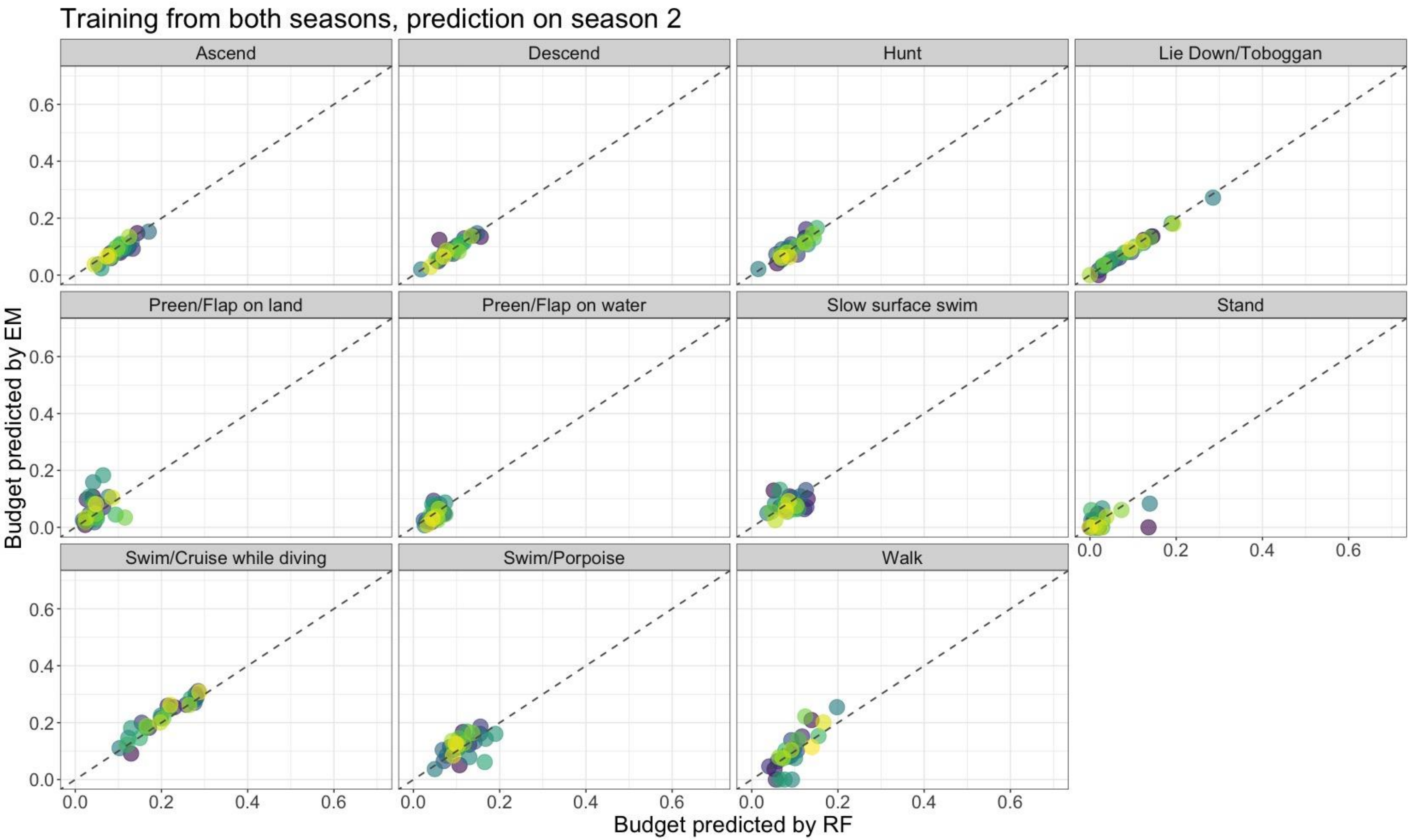

Fig. S12: Comparison between activity budgets predicted on *Season 2* by the unsupervised machine learning algorithm *Expectation Maximisation* (EM) and the supervised machine learning algorithm *Random Forest* (RF) on Adélie penguin (*Pygoscelis adeliae*). Budgets are calculated from the model runs considering training from *both seasons*. Colours indicate individual foraging trips, dashed line a theoretical 1:1 relationship.

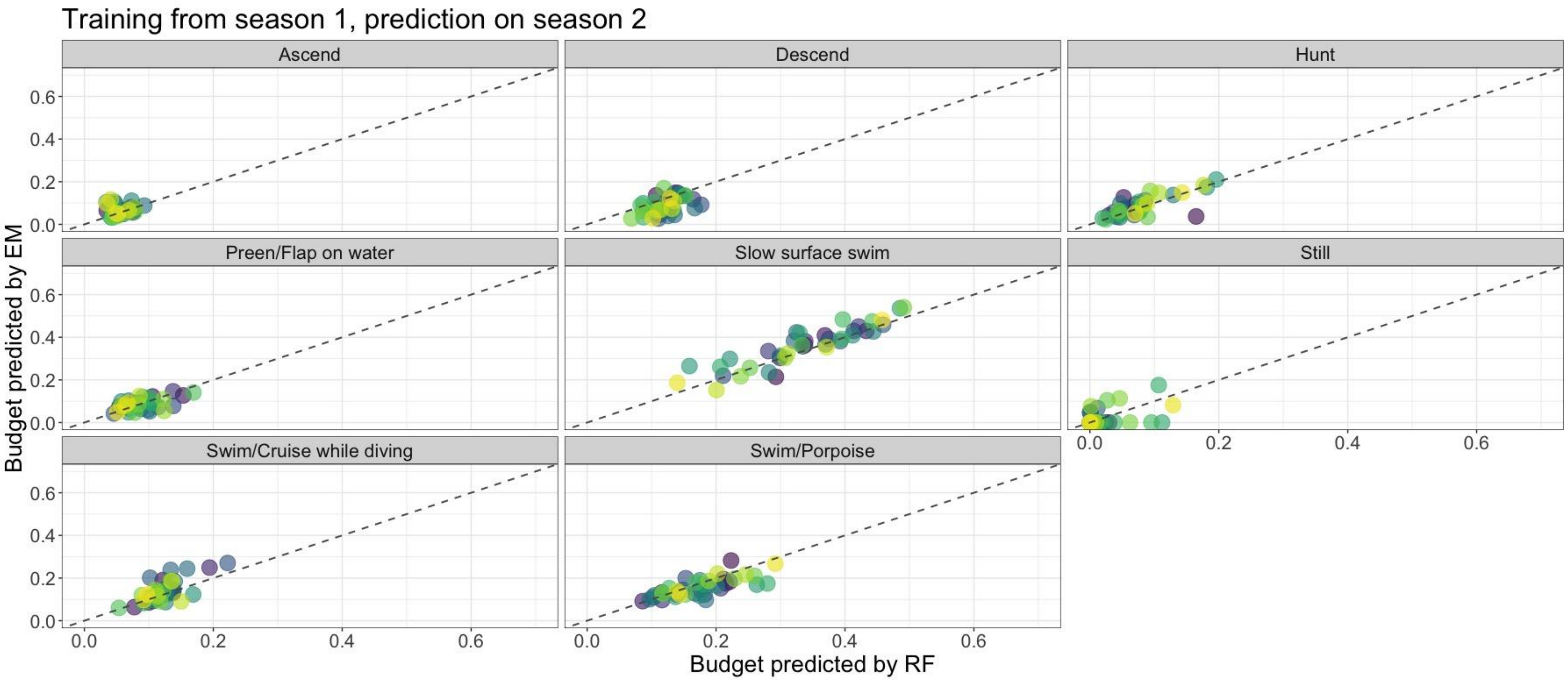

Fig. S13: Comparison between activity budgets predicted on *Season 2* by the unsupervised machine learning algorithm *Expectation Maximisation* (EM) and the supervised machine learning algorithm *Random Forest* (RF) on Little penguin (*Eudyptula minor*). Budgets are calculated from the model runs considering training from *Season 1* only. Colours indicate individual foraging trips, dashed line a theoretical 1:1 relationship.

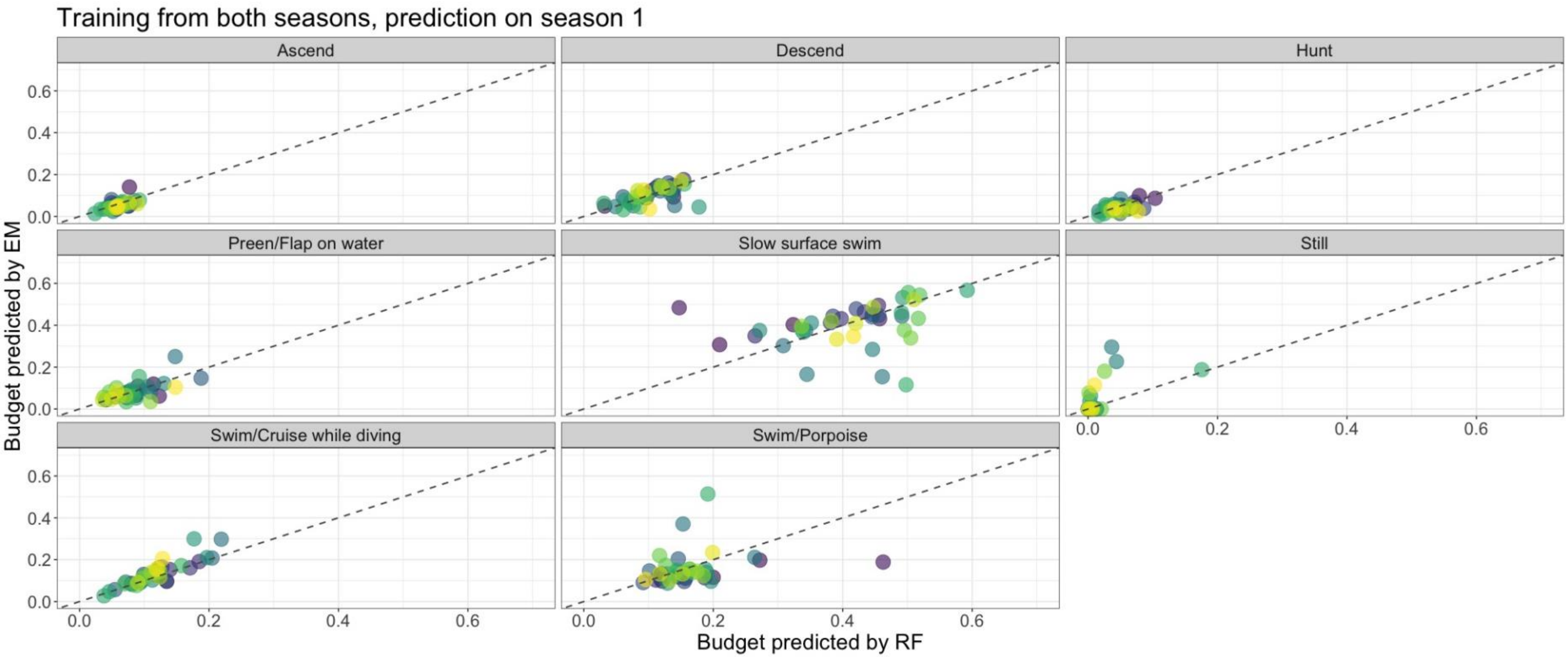

Fig. S14: Comparison between activity budgets predicted on *Season 1* by the unsupervised machine learning algorithm *Expectation Maximisation* (EM) and the supervised machine learning algorithm *Random Forest* (RF) on Little penguin (*Eudyptula minor*). Budgets are calculated from the model runs considering training from *both seasons*. Colours indicate individual foraging trips, dashed line a theoretical 1:1 relationship.

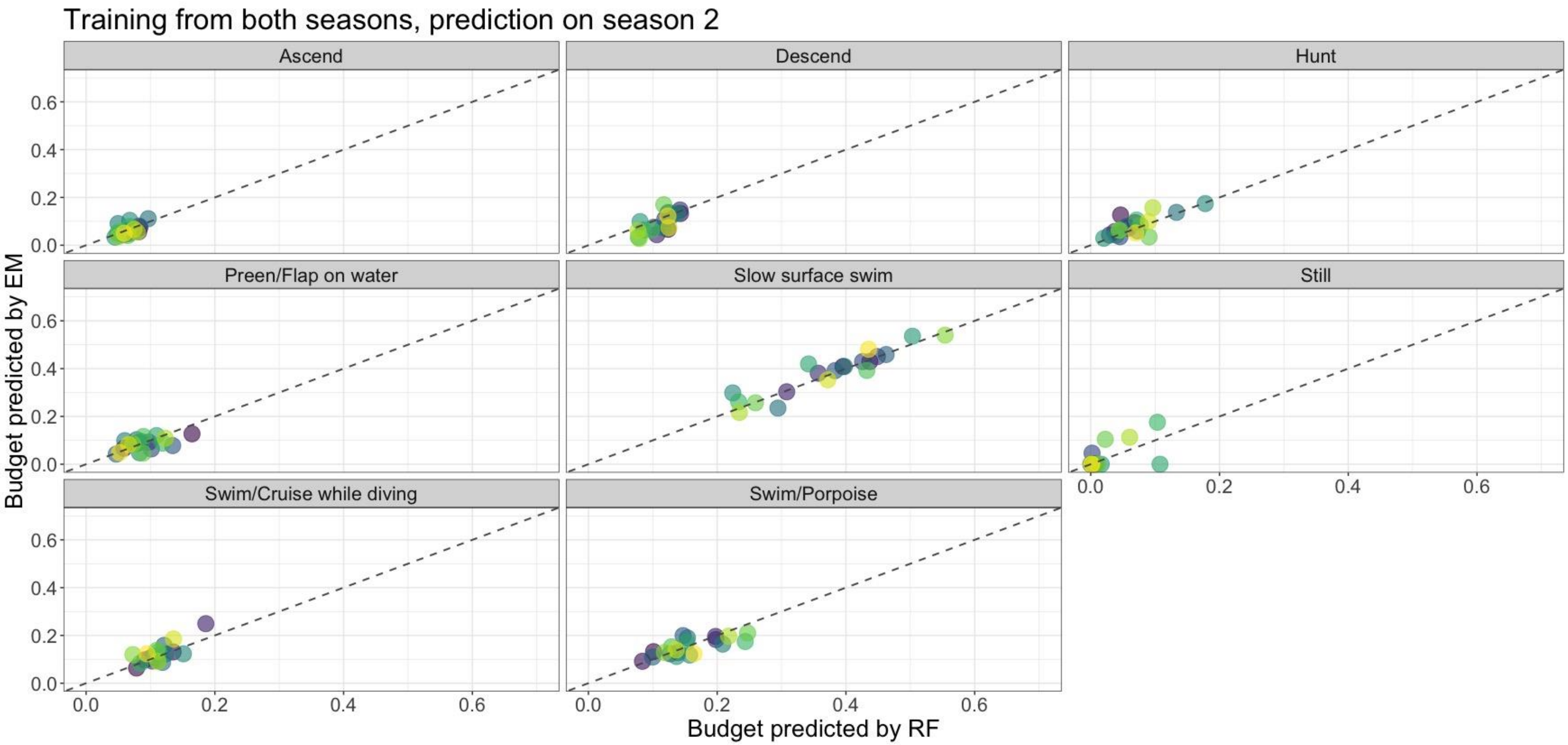

Fig. S15: Comparison between activity budgets predicted on *Season 2* by the unsupervised machine learning algorithm *Expectation Maximisation* (EM) and the supervised machine learning algorithm *Random Forest* (RF) on Little penguin (*Eudyptula minor*). Budgets are calculated from the model runs considering training from *both seasons*. Colours indicate individual foraging trips, dashed line a theoretical 1:1 relationship.

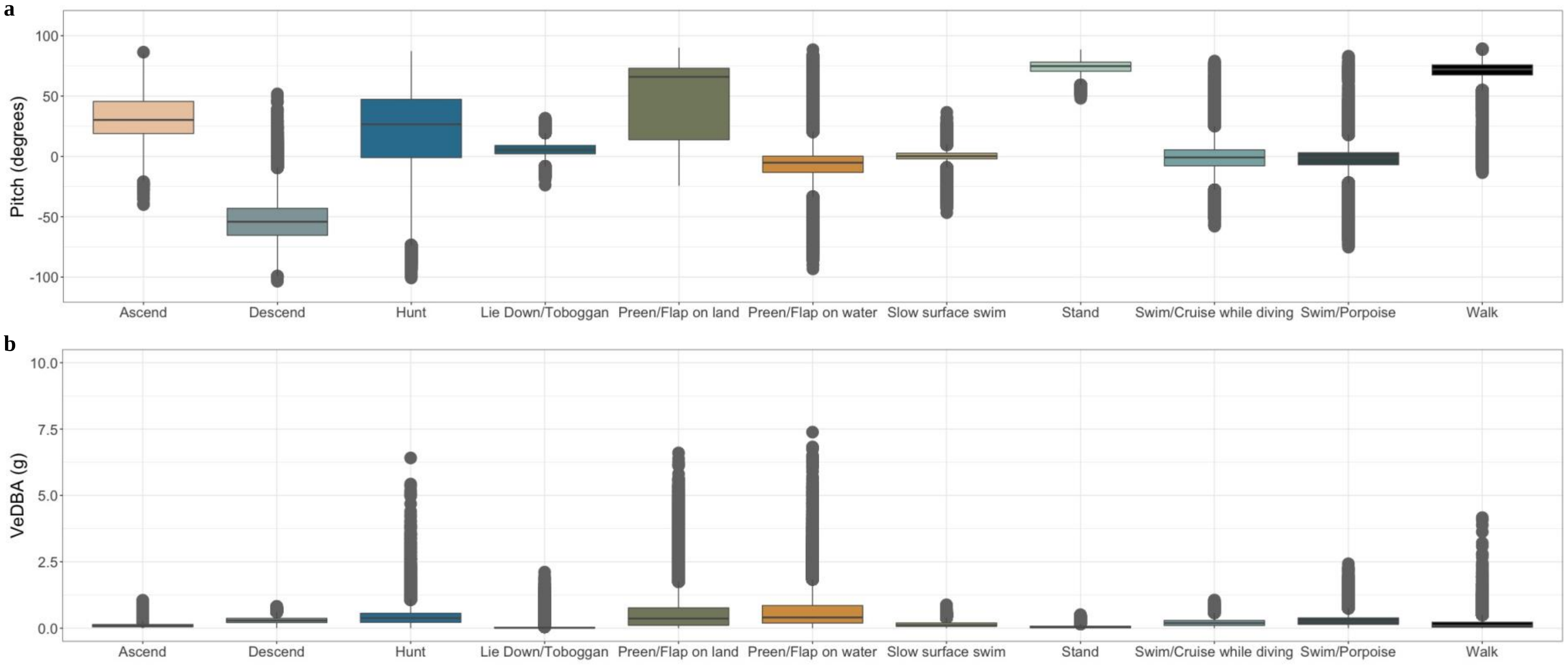

Fig. S16: Season 1 training for Adélie penguin: distribution of Pitch (degrees) and VeDBA (g) values assigned to each behavioural class for Adélie penguin (*Pygoscelis adeliae)* by the unsupervised machine learning algorithm *Expectation Maximisation* and included within the training dataset considering *Season1* only.

a

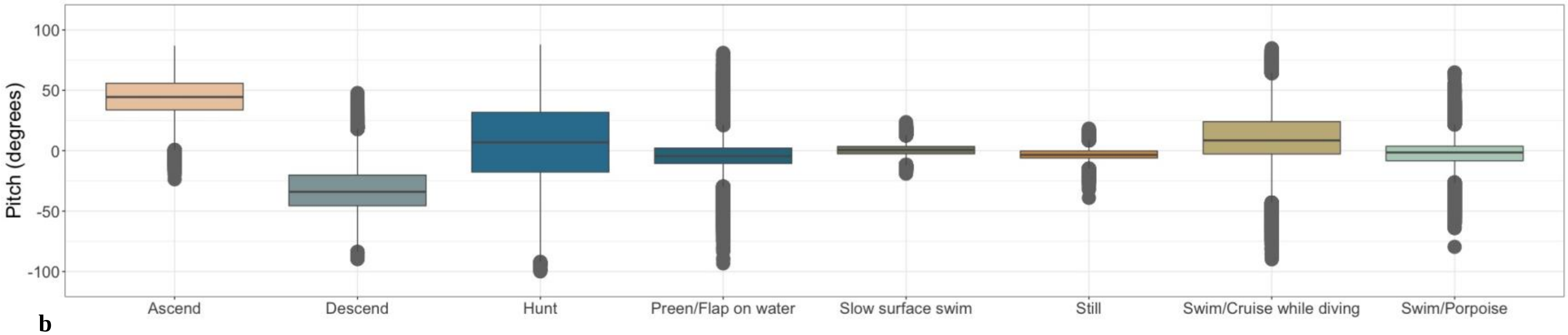

b

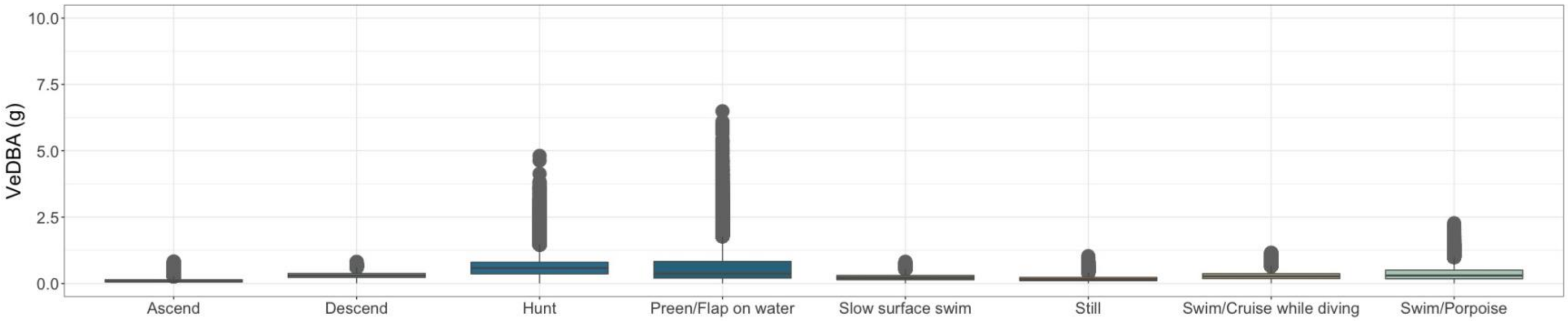

Fig. S17: Season 1 training for Little penguin: Distribution of Pitch (degrees) and VeDBA (g) values assigned to each behavioural class for Little penguin (*Eudyptula minor*) by the unsupervised machine learning algorithm *Expectation Maximisation* and included within the training dataset considering *Season1* only.

Table S1.

List of variables used to classify foraging behaviours two penguin species: Adélie penguin (*Pygoscelis adeliae)* and Little penguin (*Eudyptula minor*). Behavioural classification was performed on bio-logging data using the unsupervised classification algorithm *Expectation Maximisation* (variable selection was done through iterative approach) and the supervised classification algorithm *Random Forest* (returning values of valuable importance).

**Variable**	**Expectation Maximisation (Selection through iterative approach)**	**Random Forest (Returns variable importance)**
Depth		X
Change in Depth	X	X
Standard Deviation Heave at 2 sec	X	X
Standard Deviation Heave at 10 sec	X	X
Standard Deviation Heave at 30 sec		X
Standard Deviation Heave at 60 sec		X
Pitch	X	
Adjusted Pitch		X
Roll		X
Standard Deviation Roll at 30 sec	X	X
Standard Deviation Pitch at 60 sec		X
VeDBA	X	X
Standard Deviation VeDBA at 60 sec		X

Table S2.
Out-Of-Bag (OOB) prediction error returned by Random Forest from models run on different number of trees on both species.

OOB error	Number of trees	Species
0.10569	100	Adélie penguin
0.10381	500	
0.10351	1000	
0.10346	1500	
0.10993	100	Little penguin
0.10785	500	
0.10766	1000	
0.10761	1500	

Table S3.
Confusion matrix obtained from the Random Forest model run with 1000 trees on Adélie penguin (*Pygoscelis adeliae)*. Training dataset obtained from *Season 1*.

		Truth										
		**Ascend**	**Descend**	**Hunt**	**Lie Down/ Toboggan**	**Preen/Flap on land**	**Preen/Flap on water**	**Slow surface swim**	**Stand**	**Swim/ Porpoise**	**Swim/Cruise while diving**	**Walk**
**Prediction**	**Ascend**	72293	20	4397	0	0	0	0	0	0	4097	0
	**Descend**	3	74339	2064	0	0	0	0	0	0	1756	0
	**Hunt**	2921	972	69533	0	0	0	0	0	0	3496	0
	**Lie Down/ Toboggan**	0	0	0	64477	1115	5	422	0	79	0	336
	**Preen/Flap on land**	0	0	0	1108	64822	851	304	33	655	0	7493
	**Preen/Flap on water**	0	1	0	142	2298	70572	802	0	7844	0	9
	**Slow surface swim**	0	0	0	474	914	43	60896	0	9735	0	55
	**Stand**	0	0	0	0	43	4	0	38632	5	0	5163
	**Swim/ Porpoise**	0	0	0	760	1497	7505	13738	0	60891	0	48
	**Swim/Cruise while diving**	3940	1738	3073	0	0	0	0	0	0	69765	0
	**Walk**	0	0	0	1	6076	111	1	10027	25	0	65067

Table S4.
Confusion matrix obtained from the Random Forest model run with 1000 trees on Little penguin (*Eudyptula minor*). Training dataset obtained from *Season 1*.

		Truth							
		**Ascend**	**Descend**	**Hunt**	**Preen/Flap on water**	**Slow surface swim**	**Still**	**Swim/Porpoise**	**Swim/Cruise while diving**
**Prediction**	**Ascend**	40175	189	763	0	0	0	0	2600
	**Descend**	62	40125	1401	0	0	0	0	3715
	**Hunt**	585	757	38802	0	0	0	0	5121
	**Preen/Flap on water**	0	0	0	41379	0	1809	3460	0
	**Slow surface swim**	0	0	0	0	39887	1272	3652	0
	**Still**	0	0	0	29	368	4516	50	0
	**Swim/Porpoise**	0	0	0	2885	4118	615	36967	0
	**Swim/Cruise while diving**	3456	3260	3188	1	0	0	0	32793

Table S5.
Results of the linear model run on the Daily Energy Expenditure (DEE) calculated from time budgets estimated by both *Expectation Maximisation* and *Random Forest* methods on Adélie penguin (*Pygoscelis adeliae)* from *Season 1* and following the formula published in Hicks et al. 2020. Estimates for total energy expended during the foraging trip.

Parametric coefficients	estimate	std error	p-value	AIC	ΔAIC
(intercept)	-0.027	0.030	0.381	-296.47	169.05
DEE (by RF)	1.063	0.044	<0.001		
(intercept)	-0.041	0.013	0.001	-450.30	15.22
DEE (by RF)	1.070	0.018	<0.001		
Difference in proportion (water)	2.351	0.116	<0.001		
(intercept)	-0.043	0.012	<0.001	-465.52	0
DEE (by RF)	1.074	0.017	<0.001		
Difference in proportion (preen on water)	-2.40	0.107	<0.001		
(intercept)	-0.027	0.030	0.368	-294.92	170.6
DEE (by RF)	1.065	0.044	<0.001		
Difference in proportion (land/ice)	1.061	1.611	0.512		
**Total energy expended per trip**	**Estimate (mean±sd)**				
Total energy expended per trip (Kj) - EM	3471.5 ± 1441.636				
Total energy expended per trip (Kj) - RF	3383.9 ± 1352.533				

References

Hicks, Olivia, Akiko Kato, Frederic Angelier, Danuta M Wisniewska, Catherine Hambly, John R Speakman, Coline Marciau, and Yan Ropert-Coudert. 2020. “Acceleration Predicts Energy Expenditure in a Fat, Flightless, Diving Bird.” *Scientific Reports* 10 (1): 21493. doi:10.1038/s41598-020-78025-7.
